# Protocol for live-cell fluorescence-guided cryoFIB-milling and electron cryo-tomography of virus-infected cells

**DOI:** 10.1016/j.xpro.2022.101696

**Published:** 2022-09-22

**Authors:** Linda E. Franken, Rene Rosch, Ulrike Laugks, Kay Grünewald

**Affiliations:** 1 Leibniz Institute of Virology (LIV), Martinistraße 52, 20251 Hamburg, Germany; 2 Centre for Structural Systems Biology, Notkestraße 85, 22607 Hamburg, Germany; 3 Universität Hamburg, Institute for Biochemistry and Molecular Biology, Martin-Luther-King-Platz 6, 20146 Hamburg, Germany

**Keywords:** Cell biology, Microscopy, Molecular biology, Structural biology, Cryo-EM

## Abstract

Here, we present a protocol for assessing virus-infected cells using electron cryo-tomography (cryoET). It includes the basic workflows of seeding cells, plunge-freezing, clipping, cryo-focused ion beam milling (cryoFIB-milling), and cryoET, as well as two optional modules: micropatterning and live-cell fluorescence microscopy. We use an A549 human cell line and the virus HAdV5-pIX-mcherry in this protocol, but the comprehensive workflow can be easily transferred to other cell types and different types of virus infection or treatment.

For complete details on the use and execution of this protocol, please refer to Pfitzner et al. (2021).

## Highlights

• From growing cells on cryoEM grids to cryo-tomography on cryoFIB-milled lamellae
• Two options for preparing grids: simple coating and advanced micropatterning
• Optional live-cell fluorescence section for targeted FIB-milling

## Abstract (editor highlights)

Publisher’s note: Undertaking any experimental protocol requires adherence to local institutional guidelines for laboratory safety and ethics.

## Graphical abstract

**Figure:**
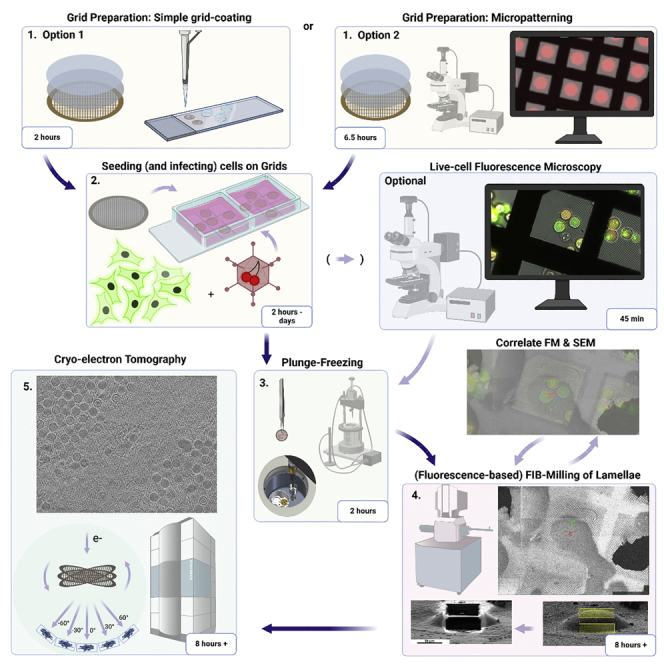

## Before you begin

This protocol describes the case of studying human adenovirus late stages of infection in A549 cells, from infection and seeding on grids to cryoET. It has been applied to determine the nuclear egress pathway of adenovirus, which starts around 48 h after infection (Pfitzner et al., 2021). However, the same protocol can be applied to study different time-points of infections, different cell lines, cells infected with different pathogens, uninfected cells and cells expressing other fluorescent reporter-proteins.

**Figure 1:**
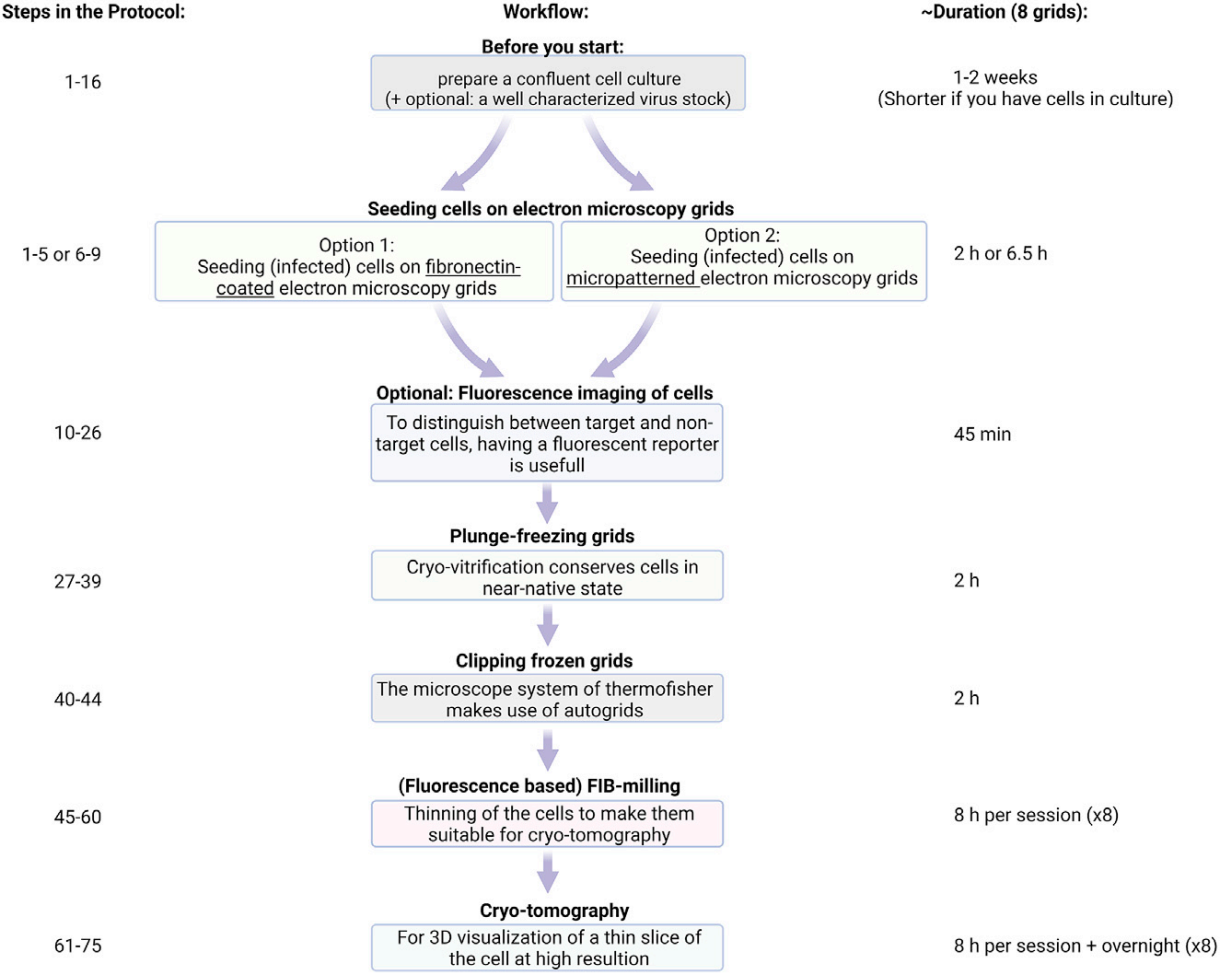
Overview of the described workflow, the corresponding steps in this protocol as well as their durations

Along the descriptions, notes can be found that include small variations, and useful handling tips, provided in a detail that stands out from a scientific paper’s methods section. Our workflow (Figure 1) is adjusted to the infrastructure available at the Advanced Light and Fluorescence Microscopy (ALFM) facility at the Centre for Structural Systems Biology (CSSB) and the CryoEM Facility at the CSSB. While fluorescence microscopy is not heavily dependent on the type of microscope, the CryoEM Facility has the electron microscopes from Thermo Fisher Scientific, which have a grid-holder system based on Autogrids. While it is expected that our protocol can be adjusted to other systems, this has not been tested and might need some adjusting.

Depending on your country’s legislation, the experiments may need to be approved by authorities prior to starting, and standard operation procedures may need to be implemented including the handling of the pathogen and of the contaminated liquid nitrogen. HAdV5-pIX-mcherry is classified at biosafety level 2 (BSL2) in Germany. The described experiments were conducted in BLS 2 laboratories, and safety measures and experiments have been approved by the government of the Hanseatic City of Hamburg before they were conducted.

Before starting with the experiment, read the full protocol, book all needed instruments and workspaces and plan carefully to assure proper timing especially between seeding and freezing, which is typically a fixed period. Plan some extra time for each step when doing them for the first time. A typical workflow would look like this: Tuesday: take an aliquot of A549 cells into culture (30 min); Thursday: split cells (30 min); Monday: split cells (30 min) and prepare electron microscopy grids (1.5 or 3.5 h); Tuesday: seed cells on grids with final step no later than 3 pm (40 min or 3 h); Thursday: image cells with fluorescence microscopy and plunge-freeze straight after, where the moment of plunge freezing is again around 3 pm to have a 48 h infection time-point (4 h but depending on number of grids). After that, grids can be stored in LN2 and planning is less restricted. Next steps are: clip grids in Autogrids (2 h); FIB-milling (1 day per grid) and cryo-tomography (1–2 days per 1–2 grid(s)).

### Preparing cells

At least one week before the experiment starts, start a cell culture of your favorite cell line (in our case A549 cells). The day before seeding cells onto the grid, split the culture once more, so that it is 70%–80% confluent the next day again. The last split assures that all cells are healthy, dividing optimally, enjoying company, and are not suffering from starvation or other issues prior to the experiment.

**CRITICAL:** Cell culture work is done under sterile conditions and is therefore performed entirely under a Class II Biological Safety Cabinet to avoid contamination.

***Note:*** Take care to keep the passage number as low as possible. Depending on the study, a passage number less than 5 times is recommended (medical or pharmaceutical applications) or in the case of our example protocol, no more than ∼30 (infection biology).

### Part 1: Taking cells into culture

**Timing: 1 week in advance; 30 min duration**

1. Preheat Growth Medium (GM) to 37°C.
2. Thaw one cryovial of A549 cells.

***Note:*** More details on this cell-line can be found at https://www.atcc.org/products/ccl-185.

***Note:*** Cells can be stored for up to 1 year at −80°C in Freezing Medium. One (1.5/2 mL) cryo-vial is filled with 1 confluent 10 cm culture dish, that was harvested, pelleted, and resuspended in 1 mL Freezing Medium. To avoid damage to the cells, the cooling rate for the freezing process is 1 ˚C/hour. This can be achieved using a Mr. Frosty chamber.

***Note:*** Quick thawing is better than slow, because cells are in contact with DMSO at toxic concentrations.

3. Transfer the cell suspension in a 15 mL Falcon tube and add 9 mL of GM.
4. Spin down for 2 min at 300 rcf.
5. Remove supernatant.
6. Add 10 mL of GM and repeat steps 4 and 5 to get in total from 10% DMSO (original cryovial concentration) to less than 0.1% DMSO, which is the amount that is no longer toxic.
7. Add 11 mL of GM to the cell pellet and change the cell suspension to a 10 cm plastic petri dish.
8. Place cells in a dedicated incubator at 37°C with 5% CO_2_.

### Part 2: Splitting cell culture

**Timing: roughly every 3–4 days, last time on the day before seeding to assure healthy growing cells; duration 30 min**

9. Preheat PBS, trypsin and GM to 37°C.
10. Check cell culture confluence with a simple light microscope.

***Note:*** Indicated amounts assume a confluent culture, seed with more volume if the dish is not confluent enough or wait one more day.

11. Work sterile. Remove medium from the cell culture dish.
12. Wash cells once with 5 mL of PBS.
13. Give 2 mL of trypsin to the dish and place in incubator for 10 min.

***Note:*** Check whether the cells are loose before stopping the reaction, some cell types require 15 min of trypsinization. If some cells have started to float, the rest can be loosened easily by pipetting some volume up and down over the dish.

14. Stop reaction with 3 mL of GM.
15. Add 0.5–1 mL (for splitting after 3–4 days) or 2 mL (for using the next day) of cell suspension to a 10 cm dish and add 10 mL of GM.

***Note:*** When using more than 2 mL of the original volume, consider spinning down (2 min at 300 rcf) and removing the supernatant to remove trypsin remains. This keeps cells healthy, stress-free and lets them attach properly in the next passage.

16. Place cells in a dedicated incubator at 37°C with 5% CO_2_.

***Note:*** The GM used in this protocol is not suitable for detailed or low-signal fluorescent imaging in the red-range due to auto-fluorescence of the phenol-red. If necessary, transfer your cells to FluoroBrite Medium (GM with FluoroBrite DMEM instead of the DMEM stated) or at least medium without phenol red. Cells may respond sensitive to a sudden change of medium. Have your cells adapt to the new medium slowly by giving a mixture of old and FluoroBrite Medium with increasing amount of FluoroBrite Medium with each round of splitting. In the case of A549 cells, one step of 50:50 (v/v) is sufficient. To avoid any effect on your experiment, allow at least one extra round of splitting in FluoroBrite Medium only.

### Preparing virus

This protocol is meant to be widely applicable and can be used to study viral infection, transfection, healthy cells, different cell types, and/or differently treated cells, and more. The preparation and characterization of virus stocks are a full workflow on themselves, and we therefore decided to place them outside the scope of this protocol. Our lab focusses on the infection cycles of viruses in the cell, and this protocol discusses mainly adenovirus infection. For the infection, it assumes that well-characterized virus preparations are already available. Prior to studying infection on grids, one should have an idea of the infection cycle and the virus stock should be titrated to determine the Multiplicity of Infection (MOI). In our example, the virus has been determined in multitude of fluorescence forming units by FACs analysis (see Pfitzner et al., 2020; 2021, for a detailed description). Here, we use the virus HAdV5-pIX-mcherry. pIX is a capsid protein located on the outside of the adenovirus capsid and in this mutant, this protein is labeled with mCherry. The signal and pattern of mCherry can be used to determine with live-cell fluorescence microscopy the stage of infection for each cell.

## Key resources table

**Table:** 

REAGENT or RESOURCE	SOURCE	IDENTIFIER
**Bacterial and virus strains**		

HAdV5-pIX-mcherry-ADP+	Pfitzner et al., 2020, 2021	N/A

**Chemicals, peptides, and recombinant proteins**		

1 M HEPES	Gibco	15630056
Dimethyl sulfoxide (DMSO)	Sigma-Aldrich, Co	D2650-5×5ML
Dulbecco’s Modified Eagle’s Medium - high glucose	Sigma-Aldrich, Co	D6546-500ML
Dulbecco’s Phosphate Buffered Saline (PBS)	Sigma-Aldrich, Co	D8537-500ML
FBS Superior	Sigma-Aldrich, Merck KGaA	S0615-500ML
Fibrinogen From Human Plasma, Alexa Fluor™ 647 Conjugate	Invitrogen - Thermo Fisher Scientific	F35200
Fibronectin	Sigma-Aldrich, Merck KGaA	F0895-1MG
FluoroBrite™ DMEM	Gibco - Thermo Scientific	A1896701
Glutamax™	Gibco - Thermo Scientific	35050-038
MEM Non-essential Amino Acid Solution (100×)	Sigma-Aldrich, Co	M7145-100ML
PLL(20)-g[3.5]- PEG(5)	SuSoS AG	https://susos.com/shop/pll20-g3-5-peg5-2/
PLPP	nanoscaleLABS	N/A
Propane/Ethane Mixture (63/37%, v/v)	Linde	special order
TrypLE™ Express with phenol red (stable trypsin Replacement Enzyme)	Gibco - Thermo Scientific	12605-010
TrypLE™ Express without phenol red (stable trypsin Replacement Enzyme)	Gibco - Thermo Scientific	12604-013

**Experimental models: Cell lines**		

A549∗	ATCC	CCL-185™
A549 lamin A/C mTagGFP∗	Pfitzner et al. (2020)	N/A
Vero E6∗	ATCC	CRL-1586™

**Software and algorithms**		

Adobe Creative Cloud	Adobe	https://www.adobe.com/de/creativecloud/plans.html
AutoTEM Cryo (2.2)	Thermo Scientific	N/A
Biorender.com	BioRender	https://biorender.com/
DigitalMicrograph Software	Gatan	https://www.gatan.com/products/tem-analysis/gatan-microscopy-suite-software
Inkscape	https://inkscape.org	N/A
Leica LASX software (3.7.0.20842)	Leica	N/A
LEONARDO v4.16 (Micromanager Plugin)	Alvéole	https://www.alveolelab.com/our-products/leonardo-photopatterning-software/
MAPS (3.17) and xT (20.1.0) user server	Thermo Scientific	N/A
Micro-Manager 1.4	Edelstein et al. (2014)	https://micro-manager.org
SerialEM (4.0.6)	Mastronarde (2003)	https://bio3d.colorado.edu/SerialEM/

**Other**		

μ-Dish 35 mm, low	ibidi GmbH	80131
μ-Slide 2 Well Co-Culture (2 × 9 well chamber slide)	ibidi GmbH	81806
1.5 mL Screw-cap tubes	Sarstedt AG & Co KG	72.692.005
10, 200, 1,000 μL Pipette and disposable tips	Eppendorf	N/A
Aquilos dual-beam FIB-SEM instrument including an upgrade to the Aquilos 2 system. (equipped with: a rotatable cryo-stage, the cryo quick loader, two in-lens and one in-column detector (trinity detection, secondary electron and backscattered electron detectors), platinum sputtering, gas injection system, MAPS software).	Thermo Scientific	N/A
Autogrid clipping station	Thermo Scientific	1000068
Autogrid loading station	Thermo Scientific	9432 909 97601
Autogrid Tweezers	Thermo Scientific	9432 909 97631
Autogrids	Custom made, Schaffer et al. (2015)	N/A
Centrifuge 5920 R	Eppendorf	5920 R
coverslip forceps for handling grids: Dumoxel 5/45	FST	https://www.finescience.com/en-US/Products/Forceps-Hemostats/Dumont-Forceps/Dumont-5-45-Cover-Slip-Forceps
Dumont N5 negative action forceps	Electron Microscopy Sciences	0103-N5-PO
Eppendorf Tubes 3810× 1.5 mL	Eppendorf	0030 125.150
Expanded Plasma Cleaner	Harrick Plasma	PDC-002
Falcon® 40 μm Cell Strainer	Corning	352340
GloQube® Plus	Quorum	N/A
HERAcell Vios 250i CO2 Incubator	Thermo Scientific	15502720
KIMTECH®Science precision wipes (low-lint tissues)	Kimberly-Clark	N/A
Leica DMi8 (equipped with: 20× air NA 0.4 objective, Lumencore Sola SE FISH 365 LED-light source, filter cubes including 480/50 nm excitation and 527/30 nm emission for green fluorescent protein (GFP) and 560/40 nm excitation and 630/75 nm emission for mCherry; with Leica LASX software)	Leica	N/A
Leica DMi8 (equipped with: HC PL FL L 20×/0.40 CORR objective, Lumencore Sola SE FISH 365 LED-light source, filter cubes including 620/60 nm excitation and 700/75 nm emission for Alexa647 dye as well a Di03-R405-t1-25×36, 405 nm laser BrightLine® single-edge super-resolution / TIRF dichroic beamsplitter for the Primo System; with Leica LASX software and Primo System from Alvéole with Leonardo Plugin in the MicroManager software)	Leica and Alvéole	N/A
Manual plunge freezer	Custom made in the Department of Biochemistry, Max Planck Institute, Martinsried, Germany. Dobro et al., 2010, Rigort et al., 2010	N/A
Maxisafe 2020 Class II Biological Safety Cabinet	Thermo Scientific	10316134 or 10325944
Microscopy slide	Thermo Scientific	SuperFrost
Mini Incubator	Labnet International	I5110A-230V
Mr. Frosty™ Freezing Container	Thermo Scientific	5100-0001
NanoCab	Thermo Scientific	9432 909 97591
Parafilm	Parafilm	N/A
PDMS (polydimethylsiloxan) stencils	nanoscaleLABS	N/A
Pipetboy acu 2	INTEGRA Biosciences	N/A
Rotor adaptor 15 mL Falcon	Eppendorf	5920757008
Rotor adaptor 50 mL Falcon	Eppendorf	5920756001
Rotor S-4×Universal-Large	Eppendorf	5895190006
Safety Cabinet Claire xl (for BSL2 plunge freezing)	Berner	N/A
Serological Pipettes 10 mL	Sarstedt AG & Co KG	36.1254.001
Serological Pipettes 5 mL	Sarstedt AG & Co KG	86.1253.001
Sputter Coater Leica ACE600	Leica Microsystems	N/A
TEM grids	Quantifoil	R2/1 Au G200F1 finder (Wash protocol 3)
TEM grids	UltrAuFoil®	R0.6/1 300 Mesh
Titan Krios 3G or 3Gi (equipped with: a X-FEG operated at 300 kV, Bioquantum energy filter and K3 electron direct detector)	Thermo Scientific and Gatan/AMETEK	N/A
Various blunt tweezers to handle cold equipment	Not specified	Similar as in: https://www.amazon.com/Tweezers-Stainless-Tweezer-Straight-Gardening/dp/B07MC65HSM
Whatman® Grade 1 Quality Filter Papers	Cytiva	1001-090

∗ Consider that passaging of cells may lead to genomic changes. Depending on your research, do not use highly passaged strains (ATCC recommends <5 for medical and biopharmaceutical applications).

## Materials and equipment

### Growth medium (GM)

Add all components into the 500 mL DMEM flask under sterile conditions.

**Table:** 

Reagent	Final concentration	Amount
DMEM	89%	500 mL
GlutaMax	1%	5.6 mL
FBS	9%	50 mL
Non-essential amino acids	1%	5.6 mL
**Total**	**N/A**	**561 mL**

When kept sterile, storage recommendations are: DMEM: 4°C; GlutaMax: 4°C; non-essential amino acids: 4°C; FBS: −20°C in aliquots of 50 mL; GM: 4°C; do not store GM or components for much longer than a year.

***Alternatives:*** When planning fluorescent experiments consider replacing DMEM for a medium without phenol-red e.g., FluoroBrite DMEM to create FluoroBrite Medium.

**Table:** Freezing Medium

Reagent	Final concentration	Amount
Growth Medium	90%	9 mL
DMSO	10%	1 mL
**Total**	**N/A**	**10 mL**

Prepare fresh right before freezing the cells.

**Table:** PLL-g-PEG Solution

Reagent	Final concentration	Amount
PLL-g-PEG	0.1 mg/mL	0.1 mg
1 M HEPES pH 7.4	10 mM	10 μL
mqH_2_O	N/A	990 μL
**Total**	**N/A**	**1 mL**

Prepare aliquots (e.g., 50 μL) and keep at −20°C; prepare in biosafety cabinet under sterile solutions; do not store for much longer than a year.

**Table:** Fibronectin Coating Solution

Reagent	Final concentration	Amount
PBS	99%	99 μL
Fibronectin	1%	1 μL
**Total**	**N/A**	**100 μL**

Prepare fresh right before coating the grids.

### Equipment

Below, we state the instruments used in our laboratory. Alternative devices can be used for the respective steps. However, the conditions and settings described are for the given instruments and might need optimization for other equipment.

### Instrument: Fluorescence microscope with photopatterning PRIMO System

(Only needed for micropatterning).

Inverted widefield microscope (Leica DMi8; HC PL FL L 20×/0.40 CORR objective, Lumencore Sola SE FISH 365 LED-light source, filter cubes including 620/60 nm excitation and 700/75 nm emission for Alexa647 dye as well a Di03-R405-t1-25×36, 405 nm laser BrightLine® single-edge super-resolution / TIRF dichroic beamsplitter for the Primo System; with Leica LASX software). Primo System from Alvéole with Leonardo Plugin in the MicroManager software.

### Instrument: Live-cell fluorescence microscope

Inverted widefield microscope equipped with a heating chamber at 37°C and 5% (v/v) CO_2_ (Leica DMi8; 20× air NA 0.4 objective, Lumencore Sola SE FISH 365 LED-light source, filter cubes including 480/50 nm excitation and 527/30 nm emission for green fluorescent protein (GFP) and 560/40 nm excitation and 630/75 nm emission for mCherry; with Leica LASX software).

### Instrument: Manual plunge freezer

Our manual plunge freezer was custom-made in the Department of Biochemistry of the Max Planck Institute (Martinsried, Germany). Images of the plunger can be found in Rigort et al. (2010) and Dobro et al. (2010). For alternative solutions, see step 27.

### Instrument: Aquilos 2 Cryo-FIB

Aquilos dual-beam FIB-SEM instrument (Thermo Fisher Scientific, Eindhoven, Netherlands) including an upgrade to the Aquilos 2 system. This upgrade allows automated lamella preparation with AutoTEM 2.2 in addition to manual preparation with the xT user interface. The system is equipped with a rotatable cryo-stage and the cryo quick loader. Installed detectors are two in-lens and one in-column detector (trinity detection, secondary electron and backscattered electron detectors). The sample can be platinum sputtered inside the chamber as well as coated with organic platinum by a gas injection system, all under cryo conditions. We also use the MAPS software package.

### Instrument: Titan Krios

Data was acquired at our 300 kV Krios 3G or 3Gi (Thermo Fisher Scientific, Eindhoven, Germany) electron microscopes equipped with an X-FEG, Bioquantum energy filter and K3 electron direct detector (Gatan/AMETEK, Unterschleissheim, Germany).

## Step-by-step method details

In this protocol, we describe all the steps needed to prepare samples for, and finally to perform cryoET on nuclear regions of HAdV5-pIX-mcherry infected A549 cells.

In the usage example, the cells are infected to study infection processes after 48 h of infection. This means that there should be (as close as possible) 48 h between the infection time-point and plunge-freezing time-point. Make sure that you time the infection sufficiently early (before or around noon), so that two days later you can also start early and have enough time for (imaging and) freezing.

**Figure 2:**
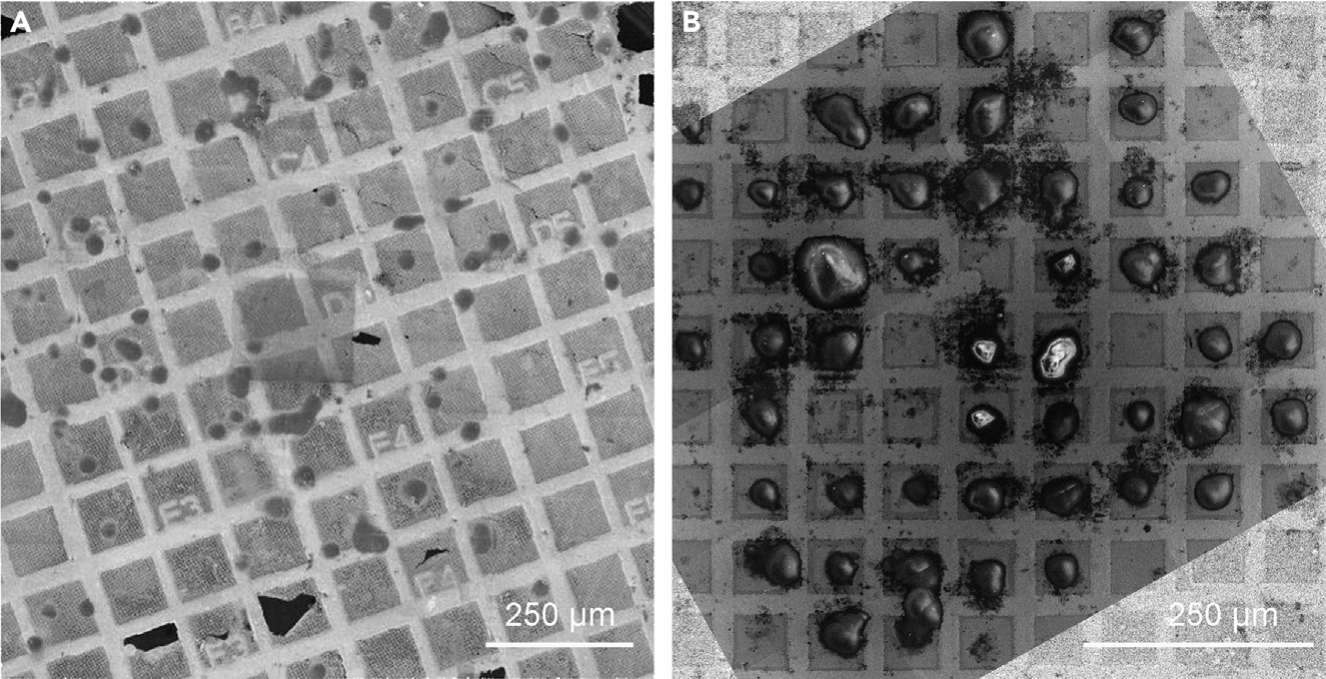
Cryo-SEM images of seeding results for a simple fibronectin coat (Option 1) and a micropatterned coat (Option 2) (A) Option 1: Seeding on fibronectin-coated grids. The cells are spread randomly and a good portion of them is sitting on top of the grid bar, inaccessible to cryoET. Shown here are Adenovirus infected A549 cells on holey carbon coated 200 mesh (R2/1) gold Quantifoil finder grids frozen 47 h post seeding. (B) Option 2: Seeding on micropatterned grids. Nearly all squares in the center of the grid are occupied by cells growing in the middle of the grid square, ideally positioned for subsequent cryoFIB-milling and cryoET. Shown here are Vero E6 cells on an UltrAuFoil R 0.6/1 300-mesh grid frozen 6 h post seeding.

Here we present two variants of grid preparation. Option 1 has been used in Pfitzner et al. (2021) and comprises a simple fibronectin coat on the surface of the grid, that allows the cells to adhere to it in a random fashion (Figure 2A). Alternatively, grids can be treated to guide cellular adherence selectively to desired areas of the grid by applying and selectively removing a cell-repelling coat. This technique is termed micropatterning (Option 2). The latter is highly advantageous, because (i) it prevents cells from clustering together, allowing a higher seeding density and (ii) it forces cells to the middle of the grid-squares, such allowing per grid many cells being optimally positioned for subsequent cryoFIB-milling (Figure 2B).

### Option 1 of preparing grids, (infecting) and seeding cells on grids: Simple coating

**Timing: 1 day before seeding, or on the day of seeding, duration 2 h**

If you would like to prepare a simple overall coating, proceed here. For the micropatterning, proceed with Option 2.

Here we make the grids suitable to grow cells on them by fibronectin coating. This renders the entire grid surface favorable and cells can randomly adhere to the grid surface.

**Figure 3:**
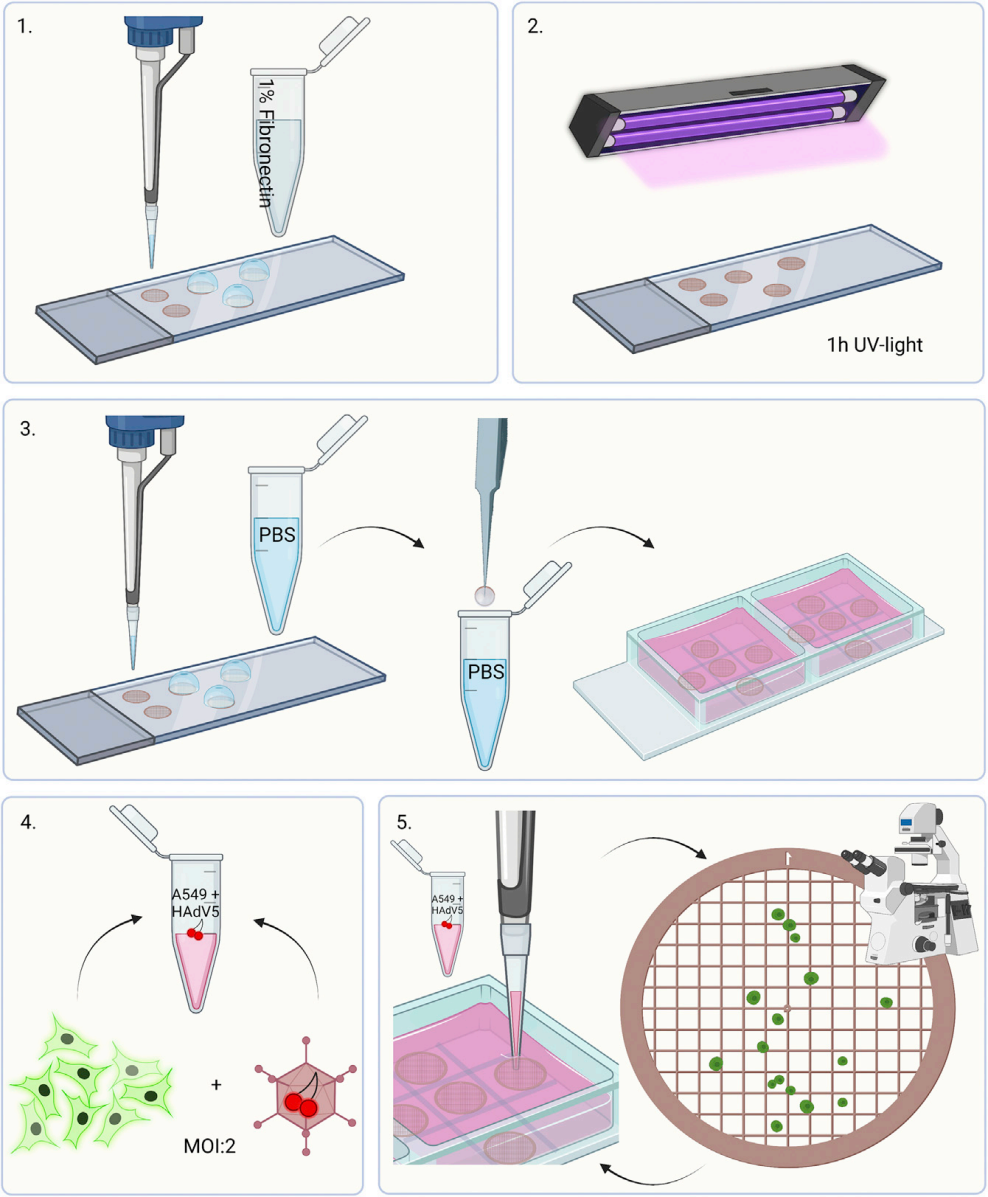
Option 1: Workflow seeding infected cells on grids 1. Apply fibronectin coating solution on glow-discharged holey carbon-coated gold finder grids (Quantifoil R2/1). 2. Let incubate for 1 h under UV-light. 3. Wet grids with PBS, wash in PBS and place in medium-filled ibidi chamber slide square. 4. Mix cells and virus at MOI:2 in Eppendorf tube. 5. Pipet small volume on grid, and check with light microscopy the cell distribution. Repeat until satisfied with the distribution.
  f. Store at 4°C or use immediately.
    **Pause point:** Sterilized grids can be stored overnight (up to 24 h) dry at 4°C and 7 days at 4°C in a wet chamber after applying a drop of PBS on them.

1. Apply the coating:
  a. Take a microscope slide and wrap with parafilm.
    ***Note:*** Parafilm is not essential but helps with the grid handling. There is no negative effect of the parafilm on the glow-discharging. However, use of parafilm is rumored to lead to pollution of the glow-discharger, which is typically not a problem for simple systems, but may lead to decreased performance of more sophisticated systems.
  b. Place the number of grids (holey carbon-coated gold finder grids (Quantifoil R 2/1)) that you would like to prepare with the foil-side facing up on the parafilm.
    ***Note:*** One batch is maximum 8 grids; 2 batches are doable and 3 are only doable when no live-cell fluorescence is applied. Depending on your experiment and proficiency, prepare 8 or 16 grids + 2 spares.
    ***Alternatives:*** Holey gold on gold grids and other grid designs can also be used to seed cells on. More sturdy grids (like the holey gold on gold) may be easier to handle.
    **CRITICAL:** Do not use copper mesh grids, as these are toxic for cells.
    ***Note:*** 200 mesh grids typically give better-positioned cells compared to smaller square sizes (like 400 mesh).
    ***Note:*** Gold-grids scatter fluorescence and it can be difficult to see a signal from your cells when the signal is not so strong. In the example protocol, this is not a problem.
    ***Note:*** Correlation of the fluorescence image to the FIB-SEM image from the FIB-SEM is more difficult with more stable grids (e.g., gold grids)*,* because the grids are undamaged during sample preparation giving less features to correlate. To help mitigate this touch each grid in two locations, away from the middle, with a sharp needle before glow-discharging. This damages 2–3 squares in each touch creating a unique pattern that helps identify the orientation of the grid. Do this before and not after seeding infected cells, because it would pose an extra safety hazard to use a needle when the material is infected.
    ***Note:*** When planning to do fluorescence microscopy, choose grids that underwent Quantifoil’s washing treatment termed “wash-protocol 3”. After reporting background fluorescence to Quantifoil, they developed several washing protocols. Wash-protocol 3 proved the best in our hands and these grids have reduced auto-fluorescence (green and red channel). This treatment can be requested when ordering grids.
  c. Glow-discharge the grids (e.g., in a GloQube) for 60 s, 25 mA, negative charge, and subsequently place the microscope slide with grids in a 10 cm glass petri dish and cover against contamination.
    ***Note:*** Even though glow-discharging works by leaking air, starting with a good vacuum seems to give a better and more complete result.
    ***Note:*** To get the grids out the glow-discharger requires air to be let in. Control the airflow to a slow setting to prevent the grids from flying around.
    ***Note:*** Use glass rather than plastic petri dishes as the latter will cause static electricity that will make your grids ‘fly’.
    ***Note:*** The effect of glow discharging wears off with time. Thus, it is recommended to proceed with the next step within an hour.
  d. Work in a safety cabinet: Prepare a fresh sterile 1% fibronectin coating solution in PBS. You need 5–10 μL final solution per grid.
    ***Note:*** The component to coat the grid with and its concentration depends on the cell type and is a parameter of optimization. Other ECM components that have been explored in our lab are laminin, fibrinogen and collagen.
  e. Put a drop (5–10 μL) of 1% fibronectin coating solution on each grid and let dry for 1 h under UV light i.e., inside the safety cabinet for sterilization (Figure 3, panels 1–2).
    **CRITICAL:** The sterilization prevents growth of bacteria or fungi and can be important for longer storage of grids, and for longer culturing of cells on the grids. It is not needed when the grids are used immediately and frozen shortly after (rule of thumb: ∼6 h). Keep grids sterile from now on.

2. Transfer of the grids into an ibidi chamber slide (2 × 9 wells):
  ***Note:*** Experienced users can save time when they do this step while incubating cells in trypsin (see step 3d), but in the beginning, the handling of grids can require more time and is easier when the user is not under time pressure.
  a. Put 10 μL of PBS on each coated grid (Figure 3, panel 3).
    **CRITICAL:** Essential before picking them up from the parafilm. This prevents tearing of the carbon layer of the grid.
  b. Prepare one ibidi chamber slide (2 × 9 wells) per experimental condition by putting 50 μL of GM in each well.
    **CRITICAL:** When incubating for longer times, take measures to prevent drying of your sample. Drying will cause increased concentrations of dilute materials, which can stress the cells and can influence the reproducibility and results of your experiments. Drying can be prevented by putting medium in all wells. The extra medium gives protection against drying of the small volumes during incubation. Alternatively, or additionally, one can place the ibidi chamber slide in a 10 cm cell culture dish and put wet lint free tissue to the side to create a wet chamber.
    ***Optional:*** FBS contains a low level of antibodies and other inhibitors. While no effect was observed for the described experiment, infection experiments at lower MOI or with other viruses could benefit from an initial infection at low or FBS free conditions. In the case of infecting, you could fill the chamber with FBS-free medium. When you do so, do not forget to carefully remove the medium from the grids and replace with (pre-warmed) GM after 1 h.
  c. Dip each grid in PBS to wash off excess fibronectin coating solution and place them one per well in a + pattern inside the ibidi chamber slide squares (Figure 3, panel 3).
    ***Alternatives:*** When working with very small volumes you can also choose to dip in GM to avoid dilution of the GM with PBS.
    ***Note:*** Leave the corners of each 3 × 3 square empty. Those corners are harder to reach with your tweezers. Grids in the middle squares are easier to grab in later steps.
    ***Note:*** Make sure the grids sink into the medium to the bottom of the dish. Having more medium helps. The order of first filling the wells with medium and then placing grids is easier, because addition of medium on top of the grids often causes them to float.

3. Harvesting cells:
  a. Preheat trypsin, PBS and GM to 37°C.
  b. Remove medium from the cell culture dish.
  c. Wash cells once with 5 mL of PBS.
  d. Give 2 mL of trypsin to the dish and place in incubator for 15–20 min.
    **CRITICAL:** Here it is important that the cells float spontaneously. If you incubate for 10 min, the cells are loose enough to rinse them off when stopping the reaction, but this often leads to cells clumping. As you need single cells, it is better to wait longer for the cells to come off spontaneously.
    ***Alternatives:*** You can also make use of a cell strainer to select for single cells.
    ***Note:*** Trypsin exposure over too long time can be harmful to the cells, so do not trypsinize much longer than 20 min.
  e. Stop trypsinization reaction with 3 mL of GM.

4. Optional: infecting cells:
  ***Note:*** For seeding cells, knowing the cell count is not needed as long as you have an 80%–90% confluent dish. In that case, continue with step 5.
  ***Note:*** When infecting cells, it is important to know the titer of the virus stock, and one needs to determine how many cells are present. For adenovirus infection, virus and cells are mixed at an MOI of 2 (as here previously determined by FACs sorting). Knowing how much virus is added is important since more virus per cell may cause the cells to progress through their infection faster, but too little virus results in less infected cells.
  a. Prepare a counting chamber (Neubauer Improved) and pipet 10 μL of cell-suspension into the chamber.
  b. Place the rest of the cells back in the incubator to keep them warm.
  c. Count all the cells in up to 4 squares under a light microscope (the more the more accurate, but if you have many cells counting 2 squares is sufficient).
  d. Calculate the number of cells per mL using the following formula:
    $$cells\;\left(ml^{-1}\right)=\frac{counted\;cells}{\#\;of\;counted\;squares}\;\times \;10^{4}$$
  e. Mix 0.5 mL of cells with virus at a MOI of 2 in a screwcap tube (Figure 3, panel 4).
    **CRITICAL:** Instead of a normal Eppendorf tube, use screw-cap tubes with a rubber seal when working with virus to avoid aerosol formation.
    ***Note:*** Make sure to store your virus in small aliquots. Typically, viruses loose infectivity by freeze-thaw cycles.
    ***Note:*** Optimize the MOI needed to get at least 80% of cells infected. It is not always possible to get 100%, since increasing the MOI too much, will increase the difference in infectivity progression between individual cells on a grid.
    ***Note:*** When having trouble with getting a proper infection, see troubleshooting 1.

5. Seeding cells on grids:
  a. Pipet 5 μL of cell-suspension and seed over one grid (Figure 3, panel 5).
    ***Alternatives:*** Seed into a square that does not contain a grid to get a first feeling of the number of cells and adjust volume from there.
    **CRITICAL:** Break the surface of the medium while pipetting instead of dropping a drop of cells. The latter will cause the cells to float more to the side and not land on the grid.
    ***Note:*** Seeding prior to infection is possible but A549 cells are fast growing. Overnight, their cell-count changes as they start to divide. This also gives rise to clumps of cells rather than single-cells, which influences the number of suitable areas for FIB-milling. Infection blocks cell division after several hours, preventing further growth.
    ***Note:*** Cells need roughly 1 h to adhere to the grid. For short timeframes, consider seeding the cells prior to infecting them, and then adding virus by pipetting above the grid, through the surface of the medium.
  b. Check the cellular distribution of the grid with a light microscope. If too few, repeat step 5a until ideally each grid square in the middle area has on average one cell.
  c. Repeat for the other grids. Discard grids with too much broken carbon.
    **CRITICAL:** The borders between wells are shallow, so do not mix different conditions in one chamber to avoid cross-contamination.
    ***Note:*** See troubleshooting 2, troubleshooting 3, troubleshooting 4, and troubleshooting 5.
  d. Put the ibidi chamber slide into an incubator and let the cells attach.
    ***Note:*** The time cells need to properly attach (and spread out for uninfected cells) is dependent on the cell line and has to be determined experimentally.

### Option 2 of preparing grids, (infecting) and seeding cells on grids: Micropatterning

**Timing: 1 day before seeding (or (in part) on the day of seeding), duration 6.5 h**

This section describes the protocol for preparing micropatterned cryoEM grids, and how to achieve a directed growth of cells on them. In this context, micropatterning describes the localized deposition of extra-cellular matrix (ECM) proteins on photo ablated areas of PEG passivated grids. One advantage of micropatterning is that one can achieve many more cells suitable for cryoFIB-milling compared to the protocol described above (Figure 3). Combined with the now established automation for lamella preparation (Klumpe et al., 2021; Kuba et al., 2021; Buckley et al., 2020; Zachs et al., 2020), this technique currently facilitates maximum throughput for the generation of cellular cryoET samples by cryoFIB-SEM (Toro-Nahuelpan et al., 2020).

**Figure 4:**
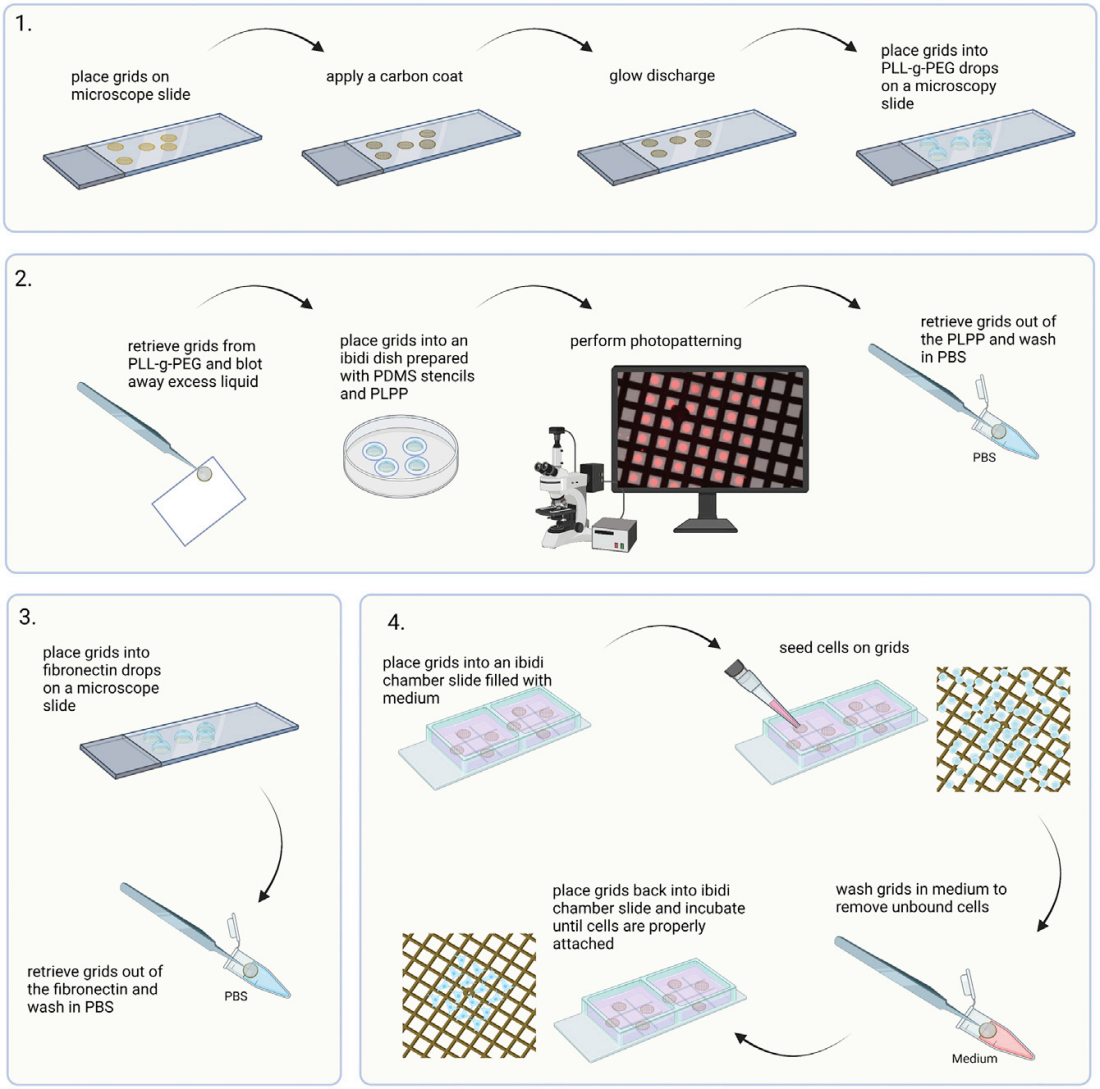
Option 2. Micropatterning workflow 1. Preparation of passivated UltrAuFoil grids. 2. Photopatterning of passivated grids. 3. Application of fibronectin. 4. Seeding cells and washing-off any out of pattern cells.

For the contactless photopatterning, we utilize the PRIMO system from Alvéole installed on a Leica DMi8 inverted microscope. Other setups have been described to create micropatterned grids (Fäßler et al., 2020; Toro-Nahuelpan et al., 2020), which may require some adaptations to the described protocol. Furthermore, the increased amount of handling steps makes the vulnerable holey carbon grids used in Option 1 (simple coating) impractical. We have good experience with more rigid grid types like gold mesh and foil grids (e.g., UltrAuFoil® R0.6/1 300 mesh) or titanium mesh (Quantifoil Micro Tools GmbH, on request) and SiO_2_ foil grids (e.g., QUANTIFOIL® R1/4 200 mesh). We advise to use reverse action tweezers (Dumont N5) for all handling steps, to prevent crushing the grid through exertion of too much force with the forceps. All handling steps in this procedure should be performed under sterile conditions where possible. An overview of the workflow is depicted in Figure 4.

**Figure 5:**
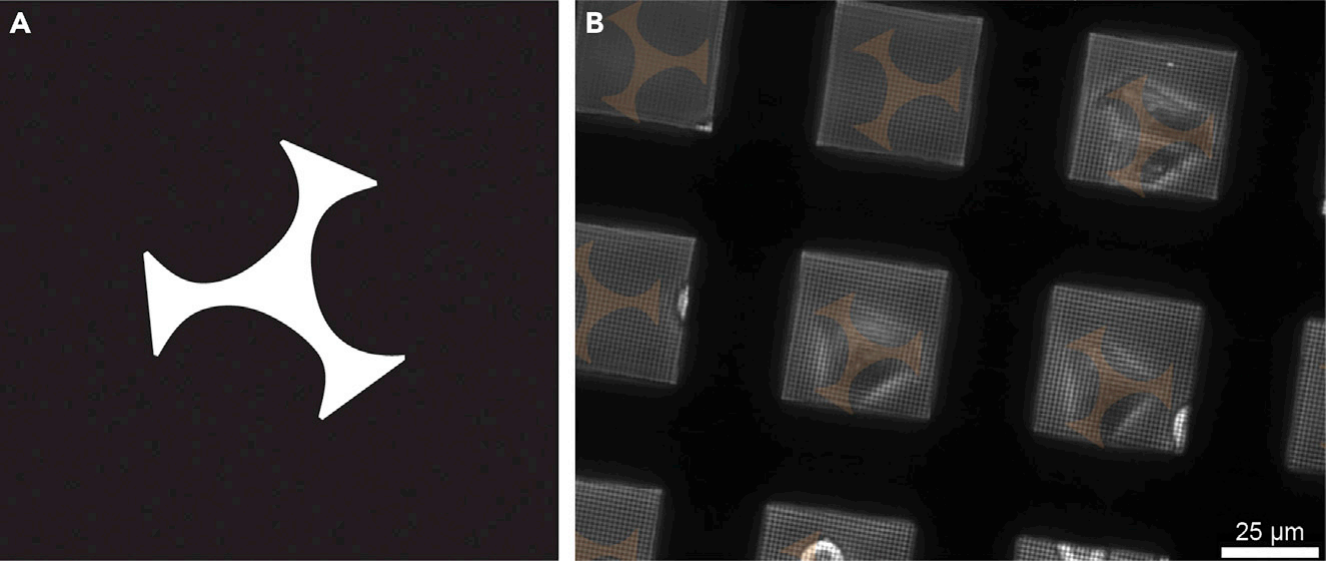
Effect of a specific pattern on A549 cells growing on a micropatterned EM grid (A) Template of the pattern used for A549 cells. (B) Transmission light microscopy of A549 cells growing on an UltrAuFoil R0.6/1 300 mesh grid passivated with PLL-g-PEG with pattern A applied (dimension: 25 × 25 μm) and coated with 10 μg/mL fibronectin. The shape of pattern A is projected onto the grid square for visualization purposes.
    vi. Set the settings to the values provided in Table 1. The selection of an ROI of 500 μm will lead to the coverage of the center of the grid with patterns.

6. Prepare grids for photopatterning:
  a. Take grids out of their box and place them foil-side up onto a microscope slide.
    **CRITICAL:** Make sure not to bend the grids. This would prevent accurate focusing during the patterning process, which is crucial for homogenous and sharp patterning results throughout the grid.
  b. Place the microscope slide into a carbon coater (e.g., Leica ACE600) and apply a 5 nm carbon coat onto the grid.
    ***Note:*** Applying a carbon coat improves the outcome of the micropatterning as well as the growth behaviour of many cell types on grids (Sibert et al., 2021).
    **Pause point:** Carbon-coated grids can be stored indefinitely.
  c. Glow discharge the grids (e.g., in a GloQube® Plus) for 60 s, 25 mA, negative charge.
    ***Note:*** The duration and intensity of the glow-discharging is a parameter that affects the effectiveness of the passivation with PLL-g-PEG (poly-L-lysine grafted with polyethylene glycol, PLL(20)-g[3.5]-PEG(5)). It is a parameter to optimize for each grid type.
  d. While glow discharging, prepare a microscope slide with parafilm and pipet a 10 μL drop PLL-g-PEG solution on it for each grid to process.
    ***Alternatives:*** A two-step passivation method using PLL and PEG-SVA can also be applied. For details, see Toro-Nahuelpan et al., 2020.
  e. Submerge the grid foil-side up into the drop of PLL-g-PEG and incubate for 1 h at RT in a wet chamber.
    ***Note:*** A wet chamber can be made from a glass petri dish containing a water-soaked lint free tissue. Do not use plastic petri dishes, as this might cause electrostatic charging.
    **Pause point:** You can also incubate the grids in PLL-g-PEG ON or for even longer periods in a fridge (4°C–8°C).

7. Perform photopatterning:
  a. Prepare a glass bottom ibidi μ-Dish 35 mm low with up to 4 PDMS (polydimethylsiloxane) stencils, which keep the grid in place during photopatterning, and add 5 μL PLPP (4-benzoylbenzyl-trimethylammonium chloride, 14.5 mg/mL) into the cavities.
    **CRITICAL:** The photo activator PLPP is toxic. Thus, appropriate PPE should be worn.
    ***Alternatives:*** You can also use a standard microscope slide to place your grids and perform the micropatterning on. However, having an enclosed chamber makes it easier to comply with sterile working techniques.
    ***Alternatives:*** Alvéole offers a PLPP-gel, which shows superior performance to the liquid one. However, its use requires a different handling and has not been tested for this protocol.
    ***Note:*** The PDMS stencil is important as it holds the grid in position and prevents grid movement due to stage movement during the patterning processes, which would result in an offset of the patterns’ location.
    ***Note:*** For a different grid positioning system than PDMS stencils, refer to Fäßler et al. (2020).
  b. Retrieve grids from PLL-g-PEG using a tweezer and carefully touch the grid’s backside with a filter paper (e.g., Whatman® Grade 1) to dry the PLL-g-PEG briefly.
  c. Immediately place the grid into the PLPP drop with the foil-side facing downwards and close the ibidi dish lid.
    ***Note:*** We are using the PRIMO system on an inverted microscope and the grids foil-side must face downwards (towards the objective) to become irradiated with UV-light. You might have to adjust the orientation of the grid according to your individual setup.
  d. Switch on the microscope including the PRIMO setup and put the ibidi dish into the microscope.
  e. Use the LEONARDO software to perform the photopatterning using the PRIMO system:
    i. Calibrate the system: First, use a highlighter and mark 5 mm on the glass in the same dish if possible. Then, follow on screen instructions.
    ii. Use bright-field illumination to put the center of the grid into the image center.
    iii. Adjust focus to the grid foil (grid appears most dark).
    iv. Click on the *magic wand icon* to open the Experimental Wizard and select *TEM Grids.*
    v. Select your pattern of interest from the pattern selection panel. For A549 cells, we found a simple triangular shape, placed in the center of the grid square, most suitable (see Figure 5).

**Table 1:** Patterning settings in the Leonardo TEM Grids Experimental Wizard

Parameter	Value
Diameter (μm)	500
Dose (mJ/mm^2^)	1,000
Pattern Size (μm)	25
    vii. Click on *Add Grid.* This will lead to the placement of patterns.
    viii. Click on *Finish* to confirm the placement of your patterns.
    ix. Click on *Play* to start the photopatterning.
      **CRITICAL:** Switch off the bright-field illumination during the photopatterning process to avoid excessive evaporation of the PLPP.
      **CRITICAL:** Only apply the pattern in well-focused areas. This is crucial for a sharp and accurate photopatterning result.
      ***Note:*** Under https://www.alveolelab.com/resources-support-center/ Alvéole provides a great resource of video tutorials and FAQs. For a detailed description of the LEONARDO software see the software’s manual, that comes with the installation.
      ***Note:*** It is advisable to look carefully at how your cell type grows in standard cell culture dishes and to adopt the dimension and shape of the cellular footprint for your pattern design.
      ***Note:*** Depending on your research question, you can use any kind of pattern to enforce e.g., cell-cell contact or specific cytoskeleton rearrangement. For examples, see Engel et al. (2021a) and (2021b); Toro-Nahuelpan et al. (2020); Théry (2010).
      ***Note:*** In our use-case, the pattern is used to force cells to grow in the middle of the grid-squares. This increases the number of cells that are in a favorable location for cryoFIB-milling.
      ***Note:*** The microscope stage performs strong shifts during the photopatterning procedure, which can lead to a shift of the grid resulting in an offset of the pattern location on the grid.
  f. Optional: You can also design your own pattern to be used in the TEM Grids Experimental Wizard of the LEONARDO software (e.g., Figure 6). Therefore, follow the following steps:
    i. Use an image editing software of your choice (e.g., Inkscape, Adobe Illustrator) and create a black square (200 px × 200 px) and place the pattern design in white (or greyscales) in the center of the black square. The pattern (white/greyscales) must have the dimension of 100 px × 100 px for the software to be able to scale it.
    ii. Save the pattern under *Program Files -> Micro-Manager-1.4 -> patterns* as an 8bit grayscale image in .tif format.
    iii. If already running, restart the LEONARDO software. The pattern should now be visible in the TEM Grids Experimental Wizards pattern selection panel.
      ***Optional:*** Instead of using the TEM Grids Experimental Wizard, you can also use a manual routine, which is described in the software’s manual.

**Figure 6:**
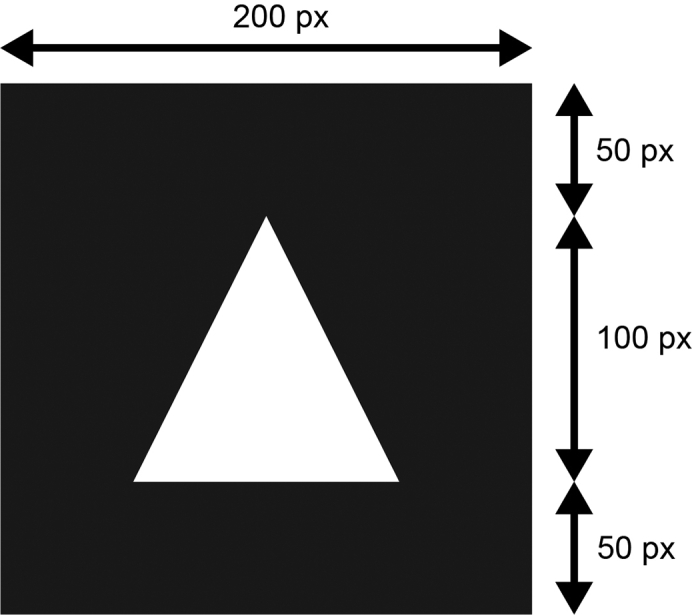
Illustration and parameters for the design of a customized pattern for the use in the TEM Grids Experimental Wizard
  g. While the pattern is applied via UV irradiation, prepare two Eppendorf tubes containing PBS, as well as a microscope slide wrapped with parafilm with a 20 μL drop of PBS for each grid.
  h. Place the ibidi dish with the photopatterned grids into the hood and carefully remove the PDMS stencil using tweezers.
  i. Pick up each grid individually and immediately dip them into the first PBS tube. Wash the grid by repeatedly (10–15×) submerging it into the PBS.
  j. Subsequently move to the second Eppendorf tube and repeat washing step.
  k. Place the grid foil-side up onto a microscope slide prepared with parafilm and PBS drops and place them into the PBS drops.
    **Pause point:** You can store the functionalized grids in drops of PBS at 4°C using a wet chamber for up to a month.

**Figure 7:**
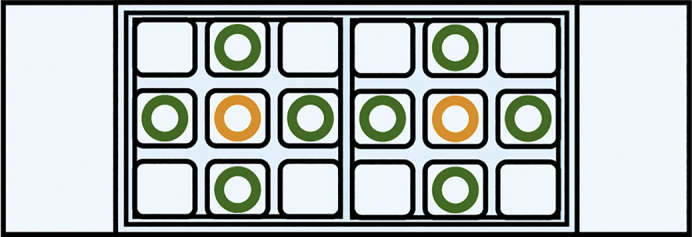
Suitable wells of a nine well ibidi co-culture chamber slide To prevent overflow into neighboring squares, only use the ones marked with a green circle. If all grids in the 9 wells have the same treatment, the squares indicated with an orange circle can also be used.
  b. Take the grids out of the fibronectin coating solution drop using your tweezer and thoroughly wash them by subsequently submerging them (10–15 times) in the prepared Eppendorf tubes filled with PBS.
    ***Optional:*** Here, you could evaluate the integrity of your pattern when having used an ECM component conjugated to a fluorescent marker/dye (e.g., Alexa647-fibrinogen). Place the grids foil-side down in a drop of PBS on a microscopy support (e.g., ibidi dish or glass slide) instead and evaluate the pattern using fluorescence microscopy (Figure 8).
    **Figure 8:**
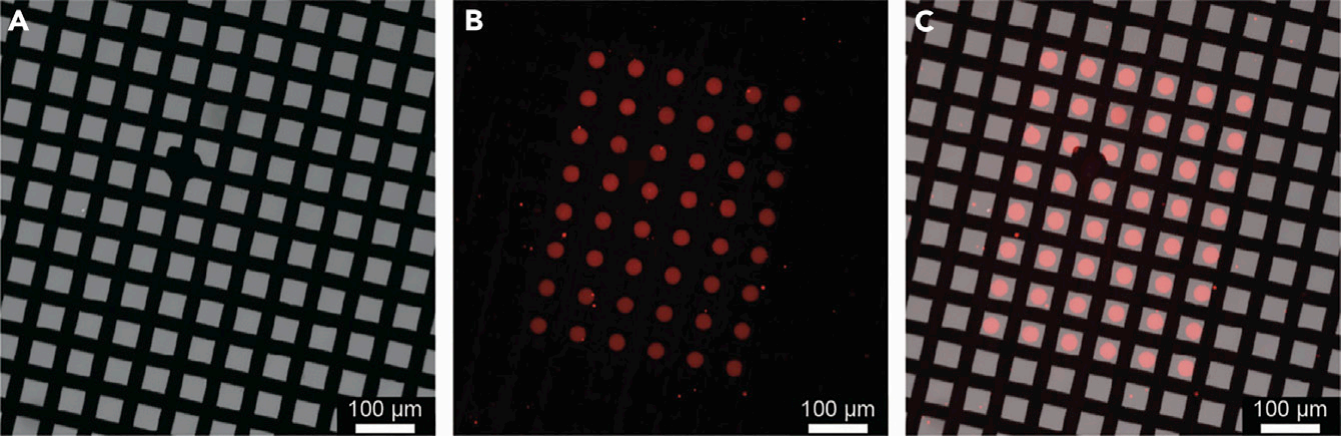
Visualization of a pattern on an UltrAUFoil 300 R0.6/1 grid coated with Alexa647-fibrinogen (A) Bright-field image of the grid. (B) Fluorescence image of the grid. (C) An overlay of (A) and (B).
  c. Place the grid foil-side up into the medium-filled wells of the ibidi chamber slide. Make sure that the grid sinks to the bottom. You can carefully submerge the grid with the help of your tweezer if necessary.
    ***Note:*** When picking up the grid out of a drop of liquid, you would like to retrieve it vertically, so that the grid retains less liquid. Be aware that your tweezer also sucks up some liquid between the forceps, which will be released upon opening the tweezers. This could dilute your media. You could also carefully blot away excess liquid from the side of the grid using filter paper.
  d. Prepare your cells now by detaching the cells in the culture flask utilizing trypsin (as described in step 3 and optional step 4).
    **CRITICAL:** It is important to stay consistent with the confluence of the cell layer at the time of trypsinization as well as the duration of the latter. Variations in these parameters will also lead to different durations for the cells to attach to the grid, which has implications for the time-point when to wash off cells from non-patterned areas of the grid. When adapting this protocol for a new cell line, it is a good start to carefully note the confluency and to keep track of the trypsinization duration (the longer the cells grow, the firmer they attach for some cell lines).
  e. Pipet your A549 cell suspension through a 40 μm cell strainer into a 50 mL Falcon tube to remove cell clumps.
    ***Note:*** Select the strainers mesh size according to your individual cell size properties.
  f. Count the cells (steps 4a–d).
  g. Prepare a dilution of 5 × 10^5^ cells/mL in GM and apply 5,000 cells (10 μL) per grid.
    ***Note:*** Pipet with the pipet tip into the medium to prevent unequal distribution of the cells onto the grid.
    ***Optional:*** Pipet 10 μL of cells into a well filled with media but without a grid to get a visual impression how many cells gives you a good coverage (1–2 cells per grid square).
    ***Note:*** Do not use less than 10 μL, as less volume results in high local concentration of the cells on your grid. Dilute the suspension with GM instead.
    ***Alternatives:*** You can prepare a 1 × 10^5^ cells/mL dilution and remove the media from the grids to then add 50 μL of cell suspension to the grid. This way you get a more equal distribution but also increase the risk of getting cells underneath the grid.
    ***Note:*** After seeding the cells onto the grid, wait 2 min to allow floating cells to settle onto the grid and check the distribution under a light microscope. If not satisfactory, add an additional appropriate number of cells.
    ***Note:*** When starting with a new cell line, it is advisable to prepare different dilutions and thus add different amounts of cells into the well with your grid.
    ***Note:*** Cells in the Falcon tube sediment rapidly. Thus, give the Falcon tube a gentle shake to homogenize the cell suspension before pipetting the cells out of the tube.
  h. To allow cells to attach to the grid, transfer the ibidi chamber slide into a 10 cm culture dish and place it into an incubator (37°C, 5% CO_2_) for 20 min.
    **CRITICAL:** Depending on the duration of the trypsinization, cell type etc. you must adjust this wait time. It is advisable to prepare a time series, e.g., 5, 10, 15, 20, 25, 30, 35, 40 min (you get 8 grids into one ibidi chamber slide), to get an impression of how long you have to wait until the cells attach properly to the grid.
  i. Retrieve the grids using a reverse action tweezer and wash off cells on the passivated areas by submerging the grid into an Eppendorf tube filled with media 10–15 times. Check the result by light microscopy.
    **CRITICAL:** Handling biologically hazardous materials with sharp tools imposes the risk of incorporation! Make sure to perform a risk assessment and to take the necessary actions to protect yourself.
    ***Note:*** The passivation is not perfect, and you will observe cells attaching to non-patterned areas of the grid. See troubleshooting 6.
    ***Note:*** When seeding cells on micropatterned grids, you are always facing the trade-off between the total coverage of patterned areas and having single cells or the desired number of cells inside the individual grid square pattern. High seeding density means a high likelihood that all your patterns will be covered, but also to obtain more than one individual cell per pattern. Choosing too low seeding densities will result in bad coverage of patterned grid squares and will result in only a few cells in patterned areas.
    ***Note:*** You aim for individual (idle) cells inside the grid square as agglomerates of cells have worse freezing properties, which will result in poorly vitrified lamellae. See troubleshooting 7.
    ***Note:*** See troubleshooting 8.
  j. Place the grid into a new ibidi chamber slide well filled with GM.
  k. Transfer the ibidi chamber slide into a 10 cm culture dish and place it into an incubator (37°C, 5% CO_2_). Wait at least 2 h for the A549 cells to flatten out and adopting the micropatterned shape.
    ***Note:*** As cells are forced through the micropatterns to grow in isolation, not every cell type will tolerate this for long. Thus, check the vitality of your cells on the grids over a time course before you start with long time course experiments (>6 h on the grid) or performing precious experiments on them.
    ***Note:*** The time cells need to firmly attach to a grid and adopt the patterns shape depends on many factors (e.g., trypsinization duration, cellular fitness, cell type, the pattern itself, etc.). Thus, it is recommended to observe on a regular basis the growth of the cells on the grid and on this basis decide when to proceed with the experiment.
    ***Note:*** If you are not satisfied with the outcome of your seeded cells on the grids, see troubleshooting 9.
  l. Perform the desired experiment and/or proceed with plunge freezing (step 27).

8. Apply an ECM component on the photopatterned grids:
  a. Prepare a microscope slide with parafilm and place a 10 μL drop of fibronectin coating solution (10 μg/mL) per grid on it.
    ***Note:*** Other cell-types may prefer other coatings to adhere to (see step 1d).
  b. Take the grids out of the PBS and place it foil-side up into the drop of fibronectin coating solution. Reassure that the grid sinks to the bottom. You can carefully submerge the grid with the help of your tweezer if necessary. Incubate at least 30 min at RT.
    ***Note:*** When picking up the grid out of a drop of PBS, you would like to retrieve it vertically, so that the grid retains less liquid. Be aware that your tweezer also sucks up some liquid between the forceps due to capillary forces, which will be released upon opening the tweezer. This could dilute your fibronectin coating solution when placing the grid into it. You can also carefully blot away excess liquid from the side of the grid using filter paper.
    **Pause point:** You can incubate/store the grids in the fibronectin coating solution drop in a wet chamber at 4°C for at least a week.

9. Seed cells on micropatterned grids:
  a. Prepare two Eppendorf tubes filled with PBS and a 2 × 9 well ibidi chamber slide and add 50 μL of warm GM into the wells except the middle and the corner ones (see Figure 7).

### Optional: Live-cell fluorescence microscopy

**Timing: 20 + 3 min per grid**

When available, live-cell fluorescence can be a great tool to indicate cells of interest for the following steps of sample preparation. In the example here, the cells are infected with HAdV5-pIX-mCherry. pIX, being one of the capsid proteins, is expressed in the infected cells and found first in the cytosol and later, when assembly starts, it is imported into the cell nucleus. The fluorescence signal can therefore indicate, even at relatively low magnifications, the stage of infection of each cell on the grid.

Note that live-cell fluorescence is not suitable as basis for accurate correlated light- and electron (cryo)-microscopy (cryoCLEM). The cells are still moving and changing in the time between imaging and freezing. It is therefore not uncommon to see differences between the locations of cells post freezing and their respective live-cell fluorescent signal. To minimize this, the timing between imaging and freezing should be kept short. When preparing more than eight grids, consider preparing them in two batches, where the first eight grids are imaged and frozen, and then the next eight grids. This also helps avoid ice-contamination as you could dry the plunge-freezer between batches and start fresh.

10. Start the microscope pc, lasers, coolers, camera, stage controller, temperature controller, CO_2_ controller and as last item the imaging software.

***Note:*** Our system is an inverted widefield microscope equipped with a heating chamber at 37°C and 5% (v/v) CO_2_ (Leica DMi8; 20× air NA 0.4 objective, Lumencore Sola SE FISH 365 LED-light source, filter cubes including 480/50 nm excitation and 527/30 nm emission for green fluorescent protein (GFP) and 560/40 nm excitation and 630/75 nm emission for mCherry) with Leica LASX software.

11. Select the configuration (*DefaultDynamicWidefielTree.xlhw + Without environmental control*).

**CRITICAL:** You want to initialize the stage; otherwise, some options are not available.

***Note:*** Environmental control can be regulated from the devices directly so no need to have this setting in the software.

12. Pre-heat microscope climate chamber to 37°C and start the CO_2_ flow into the chamber.
13. Load settings from a previous project or create new settings.

**Figure 9:**
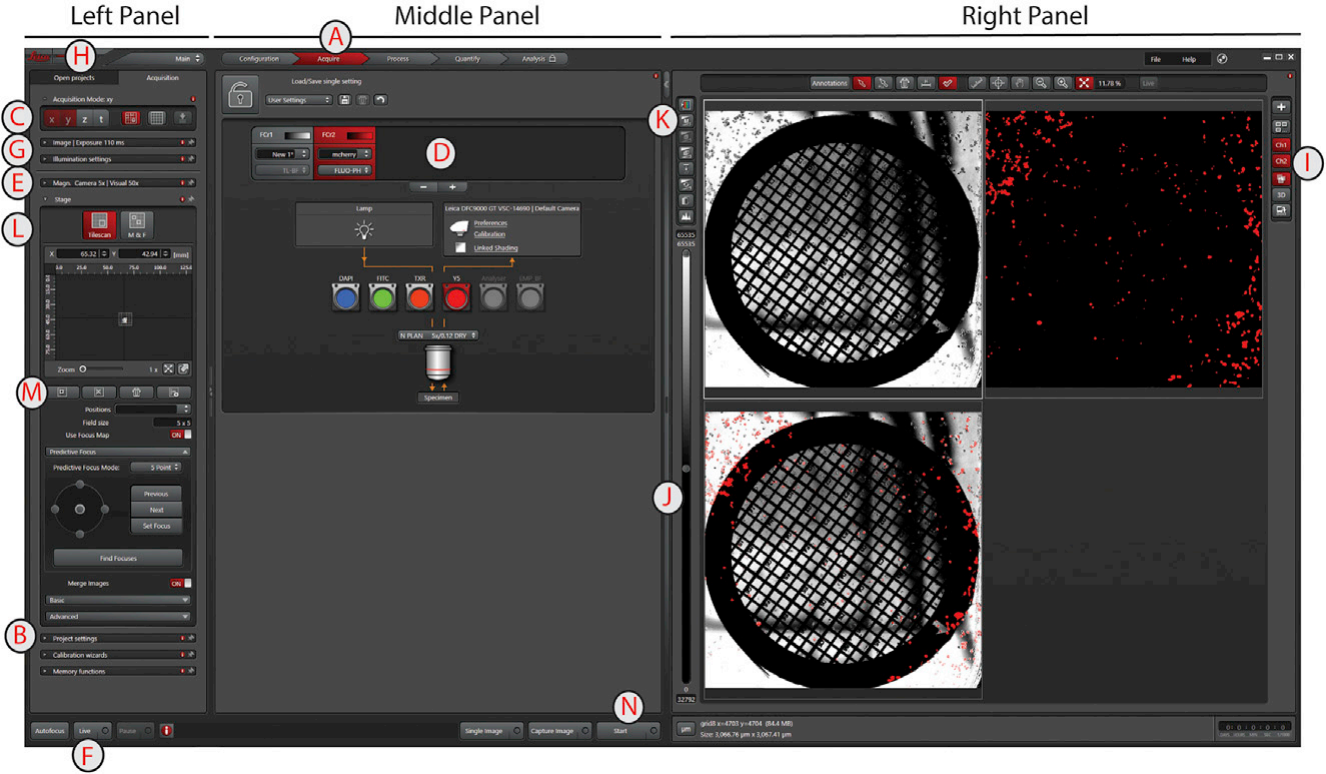
Snapshot of the Leica LASX software with panels, tabs and icons of interest marked A-N in the order they appear in the text

***Note:*** The settings can be saved and loaded in the Middle Panel under the A*cquire* tab (Figure 9A). Do not confuse them with the *Project settings* in the Left Panel (Figure 9B).

14. Set the microscope mode to single images (Figure 9C) and include one channel for brightfield, which gives you all the cells, and one channel for fluorescence, which shows the infected cells.

***Note:*** Having a bright-field image is key for a later alignment with the SEM image, because the grid will show more clearly and each cell will show in the SEM, whereas only a portion will have fluorescence.

***Note:*** In the Middle Panel at the *Acquire* tab, channels can be added or removed. Right-clicking on a new channel will allow you to pick the settings corresponding to your dye/label, in this case Leica/mCherry (Figure 9D).

15. Get the first ibidi dish with grids and give each grid a number.

***Note:*** It is important to keep track of which grid is which from now on because aligning FM to SEM can provide a challenge and guessing the grid's identity makes it unnecessarily complex.

16. Select the 5× magnifying air objective lens (Figure 9E).
17. Select the bright-field channel (Figure 9D) and click on *Live* (Figure 9F) to get a live view of your sample.
18. Center the first grid and set the z-height.
19. Change to a 20× magnifying air-objective lens (Figure 9E).
20. Set the exposure-time and laser intensity to a level that is not saturating all the signal, but that is strong enough to see the signal clearly.

***Note:*** Laser intensity and exposure settings can be changed in the Left Panel under the *Acquisition* tab in the sections *Image settings* (standard) and *illumination settings* (Advanced) (Figure 9G). Find settings suitable for your project. Here are what we have used as starting settings for the mCherry signal: image Format 2 × 2 binning and Exposure 130 ms and FIM 100%, IL – Fld. 4, Combi illumination IL and Camera 100%, and for the bright-field channel: image Format 2 × 2 binning and Exposure 95 ms Intensity 105, Aperture 24 and Camera % 100.

***Note:*** If you load a previous project (Left Panel, *Open projects* tab; Figure 9H), you can right-click on an image and click *apply image settings* to get a starting point for your imaging settings from a previous session/project.

**CRITICAL:** If you are not familiar with the microscope or the settings that you need, be careful with photobleaching. To overcome the signal reduction from the grid, laser intensities are higher than usual. Start next to the grid to find rough settings and fine-tune on the grid. The carbon film usually requires a slightly stronger laser intensity compared to cells in the ibidi dish, and gold-foil blocks a significant portion of the signal (and suffers a bit more from reflections, so carbon is more suited).

***Note:*** To inspect images, look in the Right Panel. You can select the channels that you want to see as well as the overlap to get an impression (Figure 9I). Clicking on one of the images activates the controls for that image and on the left of the images is a bar where you can adjust contrast and intensity by playing with the sliders (Figure 9J). You can also click on the icon with an “M” (or “A”) to switch between automatic and manual image settings (Figure 9K).

***Note:*** For the full potential of the viewing software, see the manual. Useful options include addition of a scale-bar and changing the colours for the channels.

21. Set up the first position using a 5 × 5 tile.

***Note:*** Even though the grid edges are not suitable for tomography, include the full grid. The edges of the grid help with alignment to the FIB-SEM image.

***Note:*** In the left panel *Acquisition* a section *Project settings* is found (Figure 9B). Project settings that we applied were *Single image mode* combined with *Z then Lambda* and *Shutter control always open*.

***Note:*** Under the *Stage* tab in the Left Panel's *Acquisition* tab, you can set the imaging mode to *Tilescan* (Figure 9L). There you can click on *Mark positions* (Figure 9M) and set the field size to 5 × 5. Switch on *Use Focus Map* and show the options for *Predictive Focus*, set the mode to *5 Point* and click *Find Focuses*. Switch on *Merge Images*, set it to *Basic* with *Auto Stitching* on *Overlap blending* Smooth and *Linear blending* on.

22. Click on *Start* to record and stitch the tiles-set for the first grid (Figure 9N).

***Note:****Single image* records a single image and *Capture image* records one image for each channel. *Start* records the full tile-set.

23. Move to the next grid. Delete the stored position by clicking the *Trash Bin* icon in the *Stage* tab of the *Acquisition* tab in the Left Panel. Start setting up a new grid (step 21).
24. Once you have finished with all the grids of the first batch, quickly move on to plunge freezing.

**CRITICAL:** Fast freezing of grids is essential to ensure minimal time between imaging and freezing for best correlation results.

25. Save the project and export the images in .tiff format.

***Note:*** The project is saved in the *Open Projects* tab in the Left Panel (Figure 9H), by activating the main project layer and licking the floppy-disk icon.

***Note:*** Stitched tiles can be exported by right clicking on the last file of the TileScan and clicking *Export Image*. It allows you to select the folder, the image type (tiff) and the channels that you would like to keep (all) and in which format. Activate *Scaled Viewer Image* and *Export Overlay as scaled Viewer Image* and click *Save*.

26. Switch off everything that you have switched on, starting with the software and finishing with the computer (or according to the local instrument protocol).

### Plunge-freezing of grids

**Timing: 1 h per 8 grids (+1 h disinfection and cleaning time afterward)**

Rapid freezing of the grids ensures the formation of vitreous ice, i.e., avoiding the formation of ice-crystals that would cause damage to the cell. Thereby, it preserves the cellular ultrastructure on the molecular level, making it possible to determine structural features to near-atomic resolution (Tegunov et al., 2021). LN2 cooled liquid ethane or the mixture of propane/ethane 63:37 (v/v) have sufficiently fast cooling rates to achieve this (Tivol et al., 2008). Mind that they also cause severe burns when in contact with skin so wear protective gear, e.g., safety glasses. When plunging at BSL2, a specialized biosafety cabinet (Model Claire XL, Berner, Elmshorn, Germany) is required! Normal biosafety cabinets have a circular airflow. This is not suitable for working with cryogenic gasses and can lead to explosive concentrations of gas. Furthermore, please consider that cryogens provide danger of suffocation. When not working under a specialized safety cabinet or chemical fume hood, make sure you are in a well-ventilated room.

27. Assemble the plunger and take a bottle of fresh and clean LN2.

**CRITICAL:** Only plungers that are adapted to allow blotting from one side (the back) are suitable.

***Note:*** Here we have used a manual gravity plunger (custom-made in the MPI of Biochemistry, Martinsried, Germany) (Rigort et al., 2010; Dobro et al., 2010). An alternative plunger that is commercially available and allows one-sided blotting is the Automatic Plunge Freezer EM GP2 (Leica). Infected cells at late stages of infection are very loosely attached to the grid and also fragile, so we have never worked with a double-sided blotter. Other labs have successfully worked with double-sided blotter like the Vitrobot (Thermo Fisher Scientific) by using a water resistant material (Teflon sheet) on the cell-facing side of the grid in combination with low blotting force (Schaffer et al., 2015).

***Note:*** Work in batches of (no more than) 8 grids per plunging session to prevent ice contamination.

***Note:*** When doing the optional fluorescent imaging (step 10), make sure to start to cool down while imaging and try to have everything cold and ready simultaneously with finishing the image of the 8^th^ grid. Waiting longer will cause ice contamination and will increase the time between imaging and freezing, leaving more room for the living cells to move around prior to being cryo-fixed.

28. Cool down the system with LN2. Avoid spilling LN2 in the ‘cup’ for the propane/ethane 63:37 (v/v).

**CRITICAL:** Working under BSL2 conditions requires wearing gloves. This poses an extra risk when working with LN2 as one is less acutely aware of the temperature changes when touching cold surfaces. Do not dip tweezers in LN2 for longer periods and be mindful of burning risks.

***Note:*** If you did spill LN2 into the ‘cup’ for liquid ethane, take a dry tissue and dip it into the nitrogen to take up the spill by capillary force. Take care with your hands and avoid touching the nitrogen for too long. Repeat with new tissue until the ‘cup’ is dry. This method is much faster compared to using warm objects to evaporate the nitrogen.

***Note:*** Wear a medical mask during all the steps that involve LN2. This will prevent the moist from your breath to cause ice-contamination and allows you to breathe freely when handling grids.

29. Cover the chamber to avoid ice-contamination (Styrofoam or paper towel) and wait for 10 min.

***Note:*** This allows everything to reach its lowest temperature. Starting too early, will make it more difficult to get a starting droplet of liquid propane/ethane in step 30.

30. Open the gas bottle and valve after the pressure valve, such that it is just before spilling gas and touch the nozzle of the tube connected to the gas bottle to the cold surface of the ethane ‘cup’. Wait a few seconds for the nozzle to cool and start a very slow gas-flow. Then fill the ‘cup’ as much as possible, i.e., until a convex meniscus is reached.

***Note:*** As soon as a small droplet of liquid has formed, the cooling rate will improve, and the gas flow can slowly be increased. Avoid excessive fogging.

31. Cover again with something light (make sure it is dry, if you use the same object), and wait for 10 min to allow the mixture to cool down.

**CRITICAL:** It is not sufficient that the gas is liquid; the temperature needs to go down as much as possible. With pure ethane, this means to wait until the edge of the container starts to show the first signs of solidification (at -182.8°C). Propane/ethane mixtures do not freeze at LN2 temperature (−196°C) but require some time to cool sufficiently.

***Note:*** We prefer the propane/ethane mix for ease of use and better freezing results.

32. Retrieve the first grid from the ibidi chamber slide.

**Figure 10:**
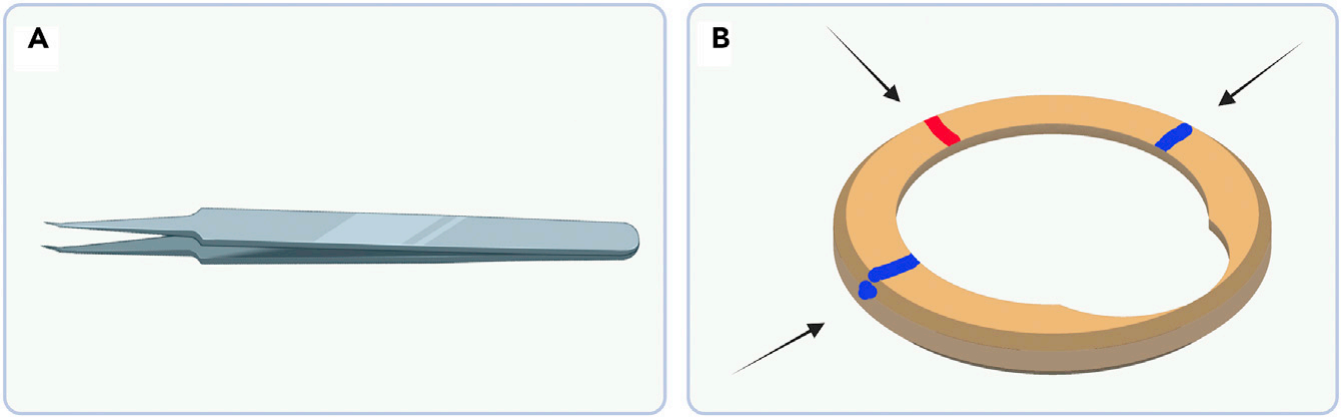
Tweezers recommendation for retrieving grids and Autogrid markings to facilitate grid orientation (A) Cover slip forceps used for retrieving grids out of the ibidi chamber slide. The bend tip helps to reach under the grid. (B) Mark the Autogrid (custom made, Schaffer et al., 2015; alternatives can be bought from Thermo Fisher Scientific: C-Clip Rings (1036173) + CryoFIB AutoGrid (1205101)) by placing a red mark across from the milling side on the flat side of the Autogrid (sometimes Autogrids are already marked, but the colored marker is much more clearly visible in liquid nitrogen) and by marking in blue each side of the wedge at 90°. With the latter mark, include the side of the Autogrid as well for easier orienting the Autogrid assembly in the Krios loading station. The red mark faces down in the FIB specimen holder and one of the blue dots faces up in the Krios cartridge.

***Note:*** This is the only step for which we do not use reverse action tweezers but cover slip forceps (Dumoxel 5/45, mind the direction of the bend, Figure 10A). Take care not to squeeze too hard to prevent damage to the grid.

***Note:*** A heating plate can be used to keep your cells at the desired temperature if no incubator is available during plunging (ideally in combination with a CO_2_ independent medium). Take care not to let the medium dry.

33. Carefully open the tweezers and place it on the bench. Grab the grid, which is now hanging lose from the tweezer-tip on your bench, over with a pair of plunging tweezers.

**CRITICAL:** Keep track of the foil-side of the grid as the grid adheres to either one of the tweezer-tips when you let the tweezer open.

***Note:*** Taking over the grid from the bend tweezers to the plunging tweezers is very hard to do without placing it first on the workbench.

34. Add a 5 μL droplet of medium on the foil-side of the grid.

***Note:*** This assures that the blotting paper gets in contact with the liquid and the additional volume significantly improves blotting reproducibility in our hands.

35. Place the tweezers with the grid into the manual plunge freezer.
36. Blot for 8 s from the back of the grid (where the cells are not sitting) with Whatman No. 1 and plunge the grids into the liquid ethane/propane.

**CRITICAL:** Blotting at the foil-side will damage the cells.

***Note:*** Blotting time is dependent on grid-type, cell density, cell type and blotting paper and may need to be optimized for your specific conditions. We experience blotting times between 4–10 s are most suitable depending on grid type and cell density.

***Note:*** Blotting too long will lead to loss of cells.

37. Place the grid in a grid box precooled in LN2 and repeat the process until all grids are frozen.

***Note:*** When working fast, 16 grids may be possible, but it is not recommended to work with a system that is cold for longer than about 30 min after cooling, to avoid ice-contamination.

***Note:*** See troubleshooting 10 and troubleshooting 11.

38. Store the grid boxes in a LN2 storage system or proceed directly with clipping.
39. Thaw the plunger setup. Be careful with the liquid ethane or ethane/propane mixture as it causes serious burns. Disinfect all surfaces that had contact with LN2.

**Pause point:** Once in LN2, the grids can be stored indefinitely. Be aware that some ice-contamination may built up in the storage over time.

### Clipping of frozen grids into autogrids

**Timing: 1 h per 8 grids (+1 h disinfection and cleaning time afterward)**

The high-end electron microscopes from Thermo Fisher Scientific™ all have holders that require the grids to be clipped into Autogrids, thus dedicated tools and a clipping station to do this.

40. Before cooling down the system, prepare the clipping tools by loading c-clips, and prepare the FIB-Autogrids by marking them (Figure 10B).

**CRITICAL:** FIB-Autogrids have a wedge in the Autogrid wall that allows milling under a shallow angle. This wedge must be oriented such that it sits at the top of the FIB-shuttle. After FIB-milling, the grid is loaded in the electron microscope at a 90° angle to have the tilt axis across the width of the lamella so that the stage can be tilted to high angles without materials coming into view. While the wedge is easily visible at room temperature, working under nitrogen conditions complicates orientation in both systems. Take a red, and green or blue marker and put a small dot on the rim of the Autogrid in the middle across of the wedge and 2 dots in a clearly distinct colour at 90° and −90° degrees from that dot (Figure 10B). Now from the Autogrid rim the orientation is easily observed.

***Note:*** Black marks are less clear and look like reflections under LN2, which is why we prefer to use colours.

41. Cool down the clipping station.
42. Transfer the first batch of grids, as well as the clipping tools into the LN2.
43. Clip the grids with cells facing down by creating an Autogrid-TEM grid-c-clip sandwich (Autogrid assembly).
44. After flipping the Autogrid assembly in the stations groove to make sure that the clip is attached properly, put them in a grid box with the wedge-marking dot (in this case red) facing down.

***Note:*** When working under BSL2 conditions, tools need to be decontaminated after having been in contact with LN2 that has contacted BSL2 samples. Time can be saved when dropping sufficient pre-marked Autogrids into the clipping station before bringing in the grids, and by having at least 4 clipping tools, preferably 8.

***Note:*** Thermo Fisher Scientific provides a manual and video material to its customers. Next to that, an impression of Autogrid clipping can be found at: Cryo-EM Grid Preparation - Assembling autogrids. In contrast to the video, we use the dedicated tweezer from Thermo Scientific™ to handle Autogrid assemblies and we flip them a few times before storing them to check the stability of the clipping as an extra precaution.

**Pause point:** Once in LN2, the grids can be stored indefinitely. Be aware that some ice-contamination may built up in the storage dewar over time.

### Lamella preparation using an Aquilos 2 Cryo-FIB

**Timing: 8 h per session, max 20 lamellae per session**

Whole cells are only transparent to the electron beam in the cell periphery. If the area of interest is deeper inside the cell, cryoFIB-milling is a mandatory step. In this process, material of the cell is ablated by a focused ion beam in such a way that only a thin lamella through the cell is left. The thickness of such lamellae is typically ∼200 nm, being thin enough to be directly transmitted by the electron beam (Marko et al., 2006; Villa et al., 2013). The procedure described here is based on an Aquilos 2 cryo-FIB system (Thermo Fisher Scientific™), which has MAPS software installed and is equipped with the AutoTEM (Kuba et al., 2021) software for automated lamella milling. Alternatively, lamellae can be milled manually or with the help of academic auto-milling software (Klumpe et al., 2021; Zachs et al., 2020; Buckley et al., 2020).

Following this protocol does not substitute instrument introduction and training as required in your microscopy facility to operate the instruments independently.

45. Cool down the Aquilos 2 loading station with LN2. Wait for minimal bubbling of the LN2.
46. Make sure grids are clipped into Autogrids (see step 40).

***Note:*** TEM grids will be clipped into Autogrids to enable safe transfer in the autoloader of the TEM.

47. Transfer 1 or 2 Autogrid assemblies from the grid storage box to the FIB/SEM shuttle taking care for correct orientation.

***Note:*** Use the mark opposite of the cut-out to orient the Autogrid assembly with cells facing up and the mark facing towards yourself. While the mark is well visible under the LN2 surface, the cut-out is hardly visible.

***Note:*** After fixing the Autogrid assembly in the shuttle, carefully and slowly flip the shuttle into loading position and check whether the Autogrid assemblies are secured properly.

48. Transfer the shuttle from the loading station into the microscope chamber with an evacuated transfer rod.

**CRITICAL:** This should take no longer than 4 min, since samples are not actively cooled during transfer and will devitrify.

49. Inspect both grids with SEM images at the lowest magnification and judge the sample quality.
  ***Note:*** Increase magnification to be able to focus the image and link the stage to working distance prior to GIS deposition.
  ***Note:*** An ideal sample would have cells in the center of grid squares and an intact grid film and cells must not be covered by a thick layer of ice but should be easily recognizable. A small amount of vitreous medium around cells should provide support and stability to the sample.
  ***Note:*** Use MAPS to take tile sets of both grids.
  ***Note:*** See troubleshooting 12, troubleshooting 13 and troubleshooting 14.
  ***Optional:*** If you have performed live cell fluorescence imaging (see step 10), you should now load the fluorescence image of your selected grid.
  a. Right-click on a new Layer in the Layer control and select *Import Images* on the context menu that appears.
  b. Find the image of your grid and open it in MAPS.
  c. Allow the program to rescale the image size if it is too big for the software.
  d. Your image appears on the right of the screen for alignment automatically.
    ***Note:*** Should you have closed this window or if you want to repeat the procedure, the alignment procedure can also be started by selecting the layer that contains your image, moussing over *Alignment* and right-clicking *Align.*
  e. Align the image by the 3-point method. This is activated by clicking the *3* behind *Points:*
  f. Find features that you can recognize in both images and place points by right-mouse clicking on the feature of image and selecting the point that you want to place.
    **CRITICAL:** The best result is obtained when the 3 points are well apart and make a triangle. Be very accurate. If you place a point again, its location is updated.
  g. Go to the next step by clicking the arrow to the right. Check the alignment and modify in the *Fine Alignment.*
  h. Click the tick-button on the bottom to complete the procedure.
50. Based on the information provided by the fluorescence data, save suitable lamella sites in MAPS.

***Note:*** Single cells should be preferred for milling, since clusters of cells are often too thick to be properly vitrified.

***Note:*** Cells on broken grid squares will not be stable enough for milling.

***Note:*** Do not mill closer than 4 grid squares away from the grid edge for a 200-mesh grid, since these positions will be out of reach for the TEM stage.

51. Apply a layer of platinum with the sputtering system (1 kV, 30 mA, 10 s), to prevent sample charging before further imaging.
52. Open AutoTEM and import the project created in MAPS.
53. Apply a milling protocol (template) suitable for the cell type. Common dimensions of lamellae are a width of 12 μm and a depth of 2 μm.

***Note:*** It is advisable to use the default template as a starting point. To create a template, which is time efficient for the sample cell size, it is advisable to monitor a full milling session with the milling currents and times for each step. By changing milling depth and correction factor, all milling times of each step will be modified at once.

***Note:*** Each step of rough, medium, fine and finer milling can further be modified separately. The goal is to set a depth correction for each milling step, which defines a suitable milling time for the step. All material within the patterns should just be milled away in the defined time. To create the most efficient milling template settings, it is advisable to monitor each milling step with a default depth correction of 100% and then modify the correction for each step. During milling, AutoTEM will provide the estimated milling time for each milling step, which is very helpful to judge if the template is suitable for the cell type.

***Note:*** When applying a template to lamella sites, it is not possible to undo and apply a new template after the preparation step.

***Note:*** A milling offset for each step will result in a more stable lamella. By applying a milling offset of 1 μm, the pattern width will be narrower with every step, creating step-shaped lamella corners.

54. Perform the preparation step in AutoTEM.

***Note:*** Depending on the sample geometry, it is advisable not to enforce the target milling-angle to prevent the preparation step from failing. Usually, a target milling-angle of 8° can be achieved.

***Note:*** If the automated eucentricity procedure is not successful, milling a mark close to the target may be helpful. Additionally, a maximal tilt step of 5° improves the eucentricity procedure. The preparation step may fail for some lamella sites. These sites might be too close to the grid front.

55. Apply an organic platinum layer with the gas injection system (GIS) onto each grid. With a deposition of 17 s, a stable protective layer of ∼4 μm is established.

***Note:*** For automated milling a thicker platinum layer than usual turned out to be beneficial.

***Note:*** The duration of the GIS deposition depends on the condition of the source and needs to be determined empirically.

56. Run the automated milling procedure without automated thinning, which we prefer to do manually.

***Note:*** For samples with ideal ice thickness around cells, automated thinning can be successful. If cells are only supported by dry film, stability tends to be insufficient for automated thinning. Thus, a thin layer of vitreous water on the support film is essential to increase the success rate of automated thinning (65%–85% as described in Tacke et al., 2021 and Klumpe et al., 2021) compared to manual thinning. As an alternative to TFS AutoTEM (Tacke et al., 2021) automated milling can be performed with SerialFIB (Klumpe et al., 2021).

***Note:*** If milling and thinning are performed automatically, select the stepwise milling order. This way the milling step is finished for all lamellae and followed by the thinning step for all sites. The thinning should always be the last action of a milling session, to minimize redeposition of material on the lamellae inside the FIB chamber.

***Note:*** For further information of automated milling and the success rate, please refer to Tacke et al. (2021).

***Note:*** AutoTEM saves the images of each preparation and milling step in the project directory. These images are very helpful when optimizing a milling protocol. Illustrations of intermediate milling steps as well as strategies for milling can be found in Schaffer et al. (2017).

57. After auto-milling is complete, lamellae are of a thickness of around 500 nm. Navigate to each lamella and perform manual thinning/polishing at a current of 30 pA.

***Note:*** Right click on *thinning position* in the AutoTEM site list and click polish position.

***Note:*** Place polish patterns into the voids, which should be 1 μm narrower than the void.

***Note:*** Some lamellae become slightly unstable, and the frontal GIS coat hangs down. In this case, only polish the top surface of the lamella.

***Note:*** The target thickness of the final lamella depends on the goals of the research. For example, a sub-tomography project may desire lamellae that are as thin as possible (below 200 nm), whereas cell-biology questions may benefit from the inclusion of more context and thus somewhat thicker lamellae (up to ∼400–500 nm).

**Figure 11:**
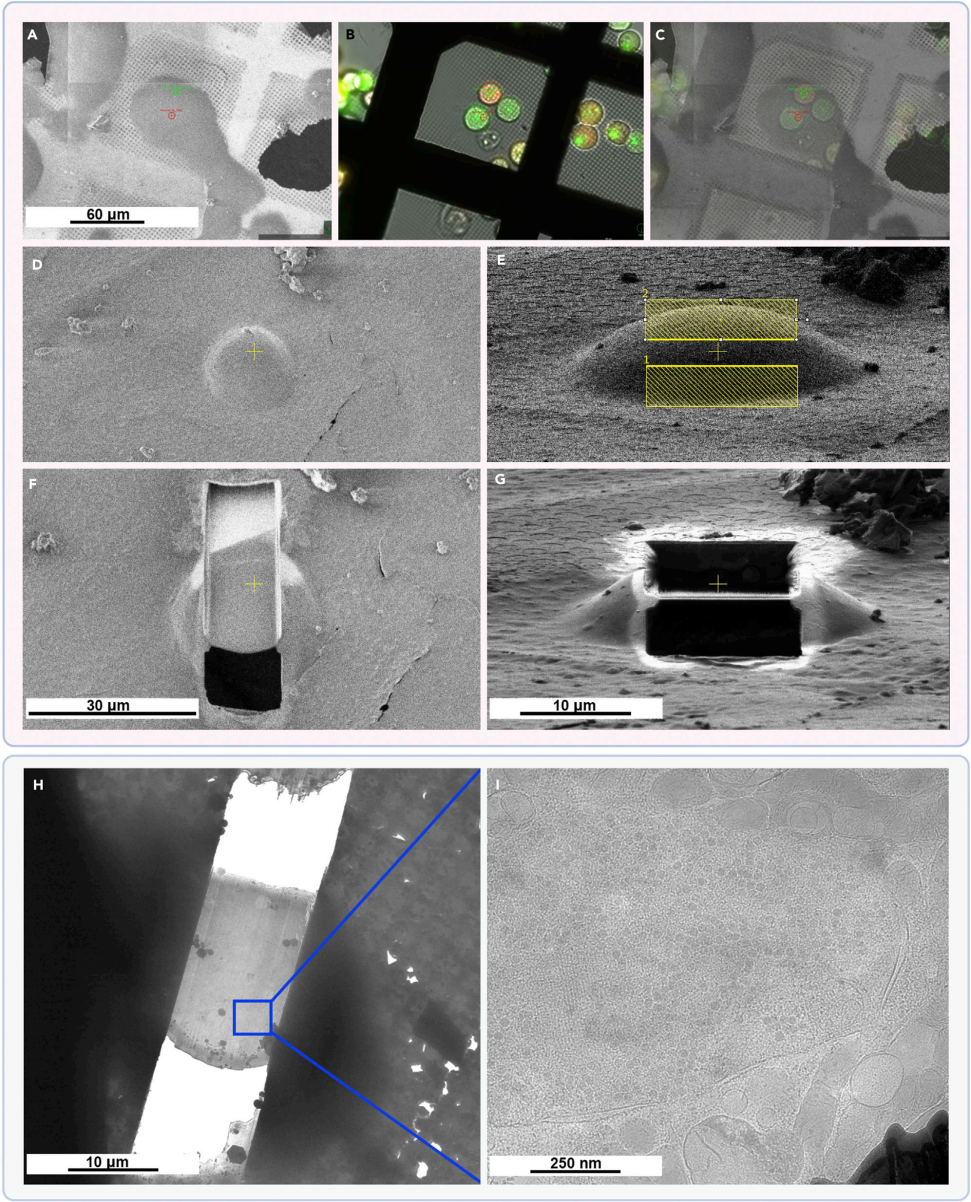
Workflow covering from the correlation of the fluorescence image with the SEM image to tomography data acquisition After loading the sample into the cryo-FIB and aligning the corresponding fluorescence image to the SEM image of the grid, sites can be selected for milling by placing milling positions. (A) The shown SEM image seems to contain one cell. (B) Fluorescence reveals that the location contains multiple cells. (C) Changing the opacity of the fluorescence image in the overlay allows pinpointing locations of interest with greater detail. (D–F) After selecting an area of interest with the scanning electron microscope, in this case an adenovirus infected human A459 lung cell (D), a pattern is drawn (E) to direct the gallium ion beam and to mill away the cellular material until a thin lamella is left (E and F). (G) Sideview of a cell after milling away the cellular material until a thin lamella is left. Panels (D) and (F) show the SEM image, and (E) and (G) the ion beam image (Dual Beam). (H) The sample is then transferred into a cryoTEM. It can be observed that only the lamella is transmitting electrons, whereas the remainder of the cell is too thick and therefore very dark. (I) A higher magnification image provides insight into the cell, showing here a part of the nucleus filled with HAdV5 virus particles. Panels D–H have been reproduced from Figure 7 from Franken et al. (2020). Fluorescence in panels A–C stems from A549 lamin A/C mTagGFP (green) and from HAdV5 pIX mCherry (red) (Pfitzner et al., 2020, 2021).

***Note:*** The final lamella should be evenly dark gray in a 5 kV SEM image (Figure 11) without stripes from insufficient polishing. To judge the lamella thickness from the SEM image, see Schaffer et al. (2017).

***Note:*** See troubleshooting 15.

58. Apply a final sputter coat to the sample (1 kV, 7 mA, 5 s).

***Note:*** This will increase conductivity for TEM imaging and is crucial to overcome charging effects during tomogram acquisition.

59. Acquire a final overview and take note of each milling angle of the individual lamellae. This information will be needed for later tomogram setup.
60. Unload the sample from the FIB-SEM microscope.

***Note:*** Avoid contamination of the sample, by using clean LN2 during unloading.

***Note:*** Aim for a fast transfer (max 4 min) of the sample from the microscope chamber to the loading station.

***Note:*** Store the sample in special Autogrid sample boxes in LN2.

**Pause point:** Οnce in LN2, the grids can be stored indefinitely. Be aware that some ice-contamination may built up in the storage over time.

### Electron cryo-tomography of lamellae with a Titan Krios TEM

**Timing: 8 h per session + 1 overnight recording per grid + 2 h wrap-up and cleaning**

Following this protocol is not sufficient to operate the Krios instrument independently. An introduction to electron cryo-microscopy in general and intense training for the specific instrument in your institute is mandatory to conduct the following experiment. Furthermore, to follow this protocol prior knowledge in SerialEM image acquisition software is needed (Mastronarde, 2003).

61. Prepare the Krios loading station by filling the station with clean LN2 in a suitable work area according to the biosafety guidelines required for the sample.
62. Transfer the sample boxes into the loading station with a pre-cooled tweezer.

***Note:*** Whenever handling samples, pre-cool tools in LN2 not too close to the sample.

***Note:*** Use dry and ice-free tools to keep the LN2 as clean as possible.

**CRITICAL:** Load the lamella sample in the correct orientation into the autoloader: lamellae must be oriented perpendicular to the tilt axis of the stage. In Krios instruments, the cut-out of the Autogrid must point to the side of the autoloader cassette, i.e., one of the blue marks should point up (Figure 10B).

***Note:*** Take note of the side the cut-out is facing or keep the side consistent. This will be critical for tomogram setup.

***Optional:*** It is advisable to load an additional grid with a carbon film, to use it for the microscope alignment.

63. Transfer the cassette into the NanoCab and dock the NanoCab to the Autoloader.
64. ‘Dock’ the cassette into the Autoloader. Safely dispose of the nitrogen from the NanoCab and disinfect it afterward.
65. Use SerialEM to acquire gridmaps at low magnification (135×) of all samples in the cassette by running the mapgrids script (Hagen et al., 2017, (https://serialemscripts.nexperion.net/script/21)).

***Note:*** Tick the option to create a map of each montage in the montage setup. Gridmaps will appear as items in the SerialEM navigator.

66. Inspect the gridmaps and assess the quality. Lamellae should appear as light gray areas in the image quality of the gridmap.

***Note:*** If lamellae are not oriented perpendicular to the tilt axis, consider reloading the grid.

67. Load the preferred grid, inspect each lamella at a medium magnification (3,600×) and high defocus and create lamella markers in the navigator (Figure 11).

***Note:*** After reloading a grid, the gridmap has to be realigned with the help of the SerialEM Navigator option “shift to marker” or “align with rotation”.

**Figure 12:**
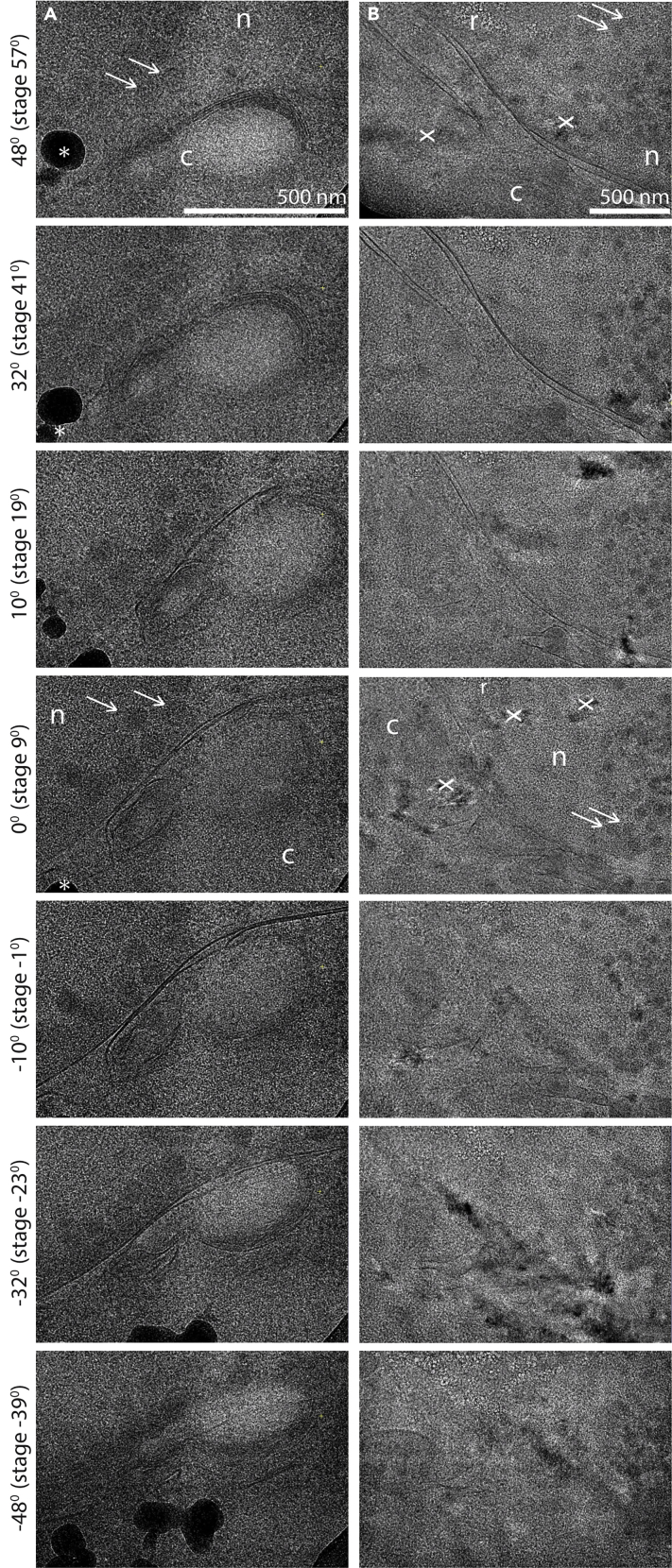
Series of selected tilt images from two unprocessed tilt series of cryoFIB-milled lamellae from Adenovirus (HAdV5-pIX-mcherry-ADP+) infected A549 cells (A) Example of a tilt series without radiation damage and with good (vitreous) ice. The reconstructed tomogram of this tilt-series can be seen in Pfitzner et al. (2021) Figure 3. (B) Example of a tilt series with radiation damage and bad (crystalline) ice. The radiation damage is likely the result of recording two tilt series in close proximity to each other leading to pre-exposure of the upper area of the displayed tilt series. These tilt series were recorded dose-symmetrically with a total dose of 110 e-/Å2 and a pixel size of 1.7 Å (A) and 3.4 Å (B) respectively. They originate from two different lamellae from the same grid. Each was recorded at the nuclear membrane of a cell, i.e., where the cell is already very thick and cryoFIB-milling was necessary to make this region of the cell accessible for cryoET. For each tilt series, the first selected tilt image as well as the tilt image recorded with a horizontally flat lamella 0° for the tomogram, but with 9° tilted stage to compensate for the milling angle, (see step 72) contain annotated features: nucleoplasm (n), cytosol (c), adenoviruses (white arrows), ice contamination on top of the lamella (∗), crystalline ice (x) and radiation damage (r).

***Note:*** Avoid acquiring data on lamellae with cracks, large ice contamination or reflections from crystalline ice (Figure 12).

***Note:*** Curtaining (caused by dense objects in the sample that led to a stripy pattern of shades) may decrease the data quality, but lamellae are still usable.

***Note:*** At this stage, it is a good time to set the eucentric height by the automatic procedure for each lamella and update the z-height of each lamella marker in the navigator.

***Note:*** See troubleshooting 16 and troubleshooting 17.

68. Decide on a magnification and defocus value for data acquisition.

***Note:*** It strongly depends on the scientific question in mind, which magnification to select. In initial steps of a project, it might be helpful to record data at a rather low magnification (e.g., 26,000×) and high defocus (-6 μm), to take advantage of the higher contrast. As soon as a specific goal is set, the magnification can be increased to fill the interested structure into the field of view of the detector. Simultaneously, the defocus should be decreased to gain resolution.

69. Check the electron dose of your data acquisition settings, considering the ideal dose for the respective detector, as well as the maximum dose the sample can take before radiation damage occurs. The K3 direct electron detector operated in counting mode performs best with a dose rate of 15 e-/px/s. Consider the dose that your sample receives and the dose that the detector needs. Tilting the sample to 60° will double its thickness. The maximum total dose cellular samples can usually withstand is ∼100 e/Å^2^.

***Note:*** The ideal beam settings are a compromise of the ideal dose rate of the detector and a minimum beam size, to prevent an overlap of the record and focus area during tomogram acquisition.

***Note:*** Depending on the platinum layer and the biological material of your sample, radiation damage may occur after very different amounts of electron dose. It is advisable to take a “bubble-gram”, when working with a new sample. This includes taking long exposure images (e.g., 3 s) and observe the onset of visible radiation damage. It is to be expected, that invisible radiation damage may take place already with much less dose. The maximum dose a sample can take is the total exposure time of the bubble-gram times the electron dose rate in e-/px/s. An example of radiation damage can be found in Figure 12B as well as Franken et al. (2017), Figure 4.

***Note:*** Calculate the exposure time of the tilt images based on the tilt scheme of the tomogram and set it in the camera setup. With a maximum dose of 100 e/Å^2^ and a typical tilt scheme (−60° to +60° with 3° increments), the dose will be spread over 41 images. Consequently, the exposure time for single images must be adapted to achieve 2.44 e/Å^2^ per image. Set the frame time to record dose fractionated data with 10 frames per image at 0° tilt. Tick the option for frame alignment or perform it during post-processing.

***Note:*** After dose calibration, SerialEM low dose mode and imaging states need to be updated.

70. Prepare the microscope for data acquisition. Depending on the system and the regulations of the microscope facility, this includes preparation of gain references and direct alignments of the beam, as well as astigmatism correction and coma correction.

***Note:*** Microscope alignments should only be performed by experienced users.

***Note:*** It is advisable to first confirm that the sample is suitable for data acquisition, before aligning the microscope to be time efficient. Thus, we recommend loading the carbon grid from the cassette to the stage at this step of the protocol to perform the alignment.

***Note:*** After reloading the sample grid, the gridmap must be realigned with the help of the SerialEM Navigator option “shift to marker” or “align with rotation”.

***Optional:*** Perform alignments on the sample grid at a spot with a thin platinum layer and skip loading a continuous carbon grid.

71. Perform a dose calibration in SerialEM, so that correct dose values will be saved in the corresponding tomogram document file (.mdoc-file).
72. Target the first spot to acquire a tomogram, refine the eucentric height, tilt the stage to a relative 0° of the lamella and take an image in data acquisition magnification.

***Note:*** To bring the lamella to relative 0°, the stage needs to be tilted to the milling angle of the respective lamella. To find out if the stage needs to be tilted into negative or positive direction, take low magnification images at −60° and +60°. Compare the size of the lamella in both images. The direction, which resulted in the larger lamella image, is the correct starting angle tilt direction.

***Note:*** During this step, observe the lamella at the extreme tilts (−/+60°) to confirm, that tomography targets are not covered by the edge of the lamella or contamination.

***Note:*** By keeping the loading direction of the cut-out relative to the autoloader cassette consistent, the starting angle will be consistent, as well. Directing the cut-out to the back of the cassette during loading, will result in a positive starting angle.

***Optional:*** At this step, it is possible to setup all targets as a batch-tomography run (Hagen et al., 2017).

73. Setup tilt series acquisition with a dose symmetric tilt scheme (Hagen et al., 2017).

***Note:*** Setup the dose symmetric tilt scheme in pairs of two. Two tilt images will be recorded before tilting to the other branch, to save time.

***Note:*** Depending on the sample thickness, it is advisable to increase the exposure time with the tilt angle, instead of keeping it constant (e.g., vary exposure time as 1/cosine to the power of ½).

***Note:*** If the stage of the system is very stable, it is not necessary to do an autofocus routine at every tilt angle. It could be reduced to every other or even less.

***Note:*** Perfect setting of the eucentric height is very critical for lamella tomography. It is consequently beneficial to repeat the refine eucentric height procedure before each tomogram.

74. Start tilt series acquisition and observe if tracking and focusing works well, by paying attention to the focus values in the SerialEM log file.
75. After all tilt series are acquired, finish the session with unloading the sample from the stage and autoloader.

***Note:*** Discard all LN2 as well as the samples according to the biosafety regulations suitable for the pathogen used.

***Note:*** Clean all the material, which had contact to the sample according to the biosafety regulations suitable for the pathogen used.

Congratulations!

You acquired a tilt series of the lamella prepared from an infected cell.

We advise to perform the following inspection of data and tomogram reconstruction with the IMOD software package (Mastronarde and Held, 2017, https://bio3d.colorado.edu/imod/). Additional to data reconstruction, the package includes functions for tomogram segmentation and visualization. While there are many alternative image processing packages emerging, IMOD is ideal to get started.

## Expected outcomes

Figure 11 shows the expected outcome, as well as some intermediate results. When the workflow goes well, the cells have not moved between live-cell fluorescence imaging and plunge-freezing (Figures 11A–11C) and the fluorescence can be used to guide the FIB-milling (Figures 11D–11G), which is ideally performed on cells that sit close to the center of the grid and in the middle of a grid square, as is the case in the example. After transferring the grid to the Krios, the lamella can be located, and images and tomograms can be recorded of the interior of the cell (Figures 11H and 11I).

In Figure 12, two sets of images from tilt series recordings on a lamella are shown. While Figure 12A illustrates the desired outcome for a tilt series, Figure 12B shows one that has been recorded in an area of poor vitrification and is of limited use (see also limitations).

## Limitations

While the fluorescence can help locate cells of interest, it is not going to be accurate enough to locate sub-cellular features. If the site of interest is very small and hard to locate, consider using actual cryo-CLEM, which requires a fluorescence microscope with a cryo-stage and implies a more elaborate workflow (Schorb et al., 2017; Schellenberger et al., 2014). It increases the chance of ice-contamination, but also allows much more accurate targeting.

A further limitation is posed by the thickness of the cell. Cells that are infected with Adenovirus become round and even thicker than healthy well-spread cells. In general, human cells are on the border-thickness of what can be successfully frozen into vitreous ice. Therefore, you will often encounter lamellae and tomograms that include patches of crystalline ice (see troubleshooting 16). Figure 12 illustrates this issue. It should be considered that the specimens’ ultrastructure in areas with crystalline ice has been damaged by the freezing process and care should be taken when interpreting such data.

## Troubleshooting

### Problem 1

Cells show only poor infection (step 4).

### Potential solution

• Give the infection time before continuing with seeding and incubate the cells and virus prior to seeding.
• Increase the MOI.
• Rather than increasing the MOI, consider working in FBS-free medium. FBS contains small amounts of antibodies and could interfere with cellular infection. Not only put FBS-free medium in the ibidi chamber slides, but also remove it from the harvested cells before adding virus to the cells. This is done by spinning down a volume of counted cells and resuspending them in FBS-free Medium before adding virus. After 1 h of infection (on the grid), the virus should have entered the cells and the medium needs to be replaced with FBS containing medium. In this case, do not wait 1 h before seeding cells because cellular adherence is negatively affected by infection.

### Problem 2

Cells do not adhere to simple functionalized grids (step 5).

### Potential solution

• Try a different coating reagent e.g., poly-L-lysine.
• Your grid might have some contaminations left from the production process that impact on cell viability. Wash your grids with isopropanol or ethanol and subsequently with water to get rid of potential remnants.

### Problem 3

Cells are solely growing on the grid bars (step 5).

### Potential solution

• Use carbon foil grids. Some cell types prefer only stable surfaces to adhere to. Consider using a different grid type.
• The coating reagent can influence the cells tendency to aim for the grid bars. Try different reagents to optimize the coat for your cells.
• For the best results, try micropatterning.

### Problem 4

Many grid squares are broken and/or the grid is bend (step 5).

### Potential solution

• Handle grids with care. Check if your tweezers tip is free from damage. You can use a simple light microscope for inspection or carefully listen when opening and closing the tweezer. There should be no noise.
• Use negative action tweezers where possible to prevent the exertion of too much force to the grid.
• Only grab the grid using a sharp tweezer on the edge. Avoid grabbing onto the foil.
• Choose a more stable grid-type. We have good experience with gold mesh and foil grids (UltrAuFoil® from Quantifoil) or Titanium mesh and SiO_2_ foil grids.
• Carbon grids can get damaged when letting too much air into the glow-discharger too fast. Vent the vacuum chamber slower.

### Problem 5

You observe mainly cell clumps on your simple coated grids (step 5).

### Potential solution

• Use a cell strainer to select for single cells.
• Passage your cells at a lower confluence.
• Adjust the concentration of trypsin and the incubation time for detachment.
• Decrease the incubation time of the cells on the grid to prevent the cells from dividing, which leads to agglomeration.

### Problem 6

**Figure 13:**
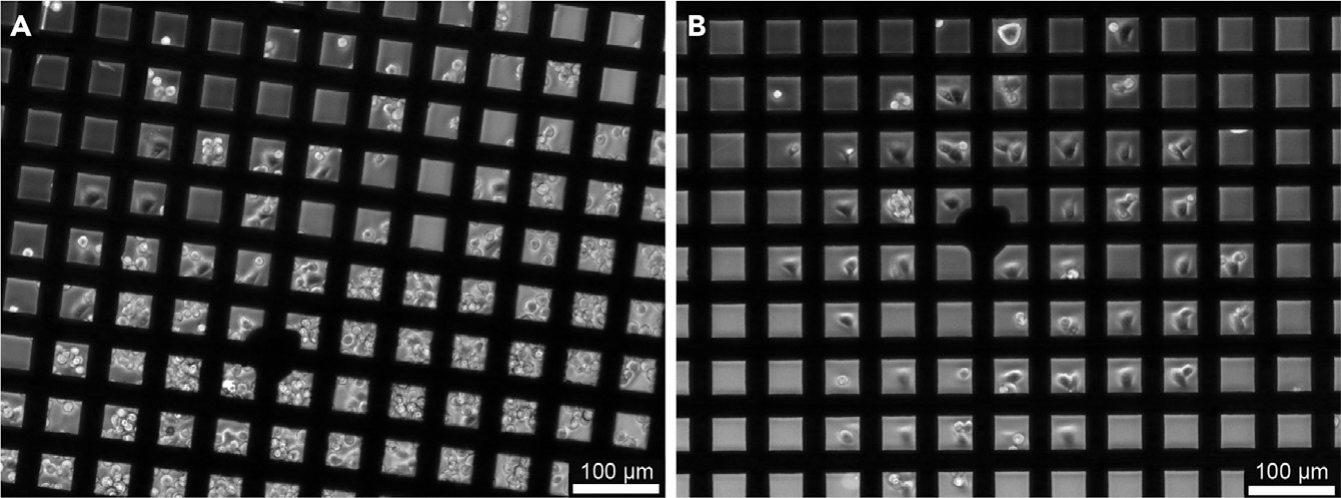
Unsuccessful vs. successful outcome of A549 cells seeded on micropatterned UltrAuFoil R0.6/1 300 grids shown in a bright-field light microscopy image (A) High local concentration of cells during seeding leads to multiple cells in a grid square. Cell agglomerates can’t be washed off anymore. This grid is likely to be unusable as the dense cell layer interferes with proper blotting and freezing. (B) Desired outcome of cultivating cells on micropatterned grids. Most cells are single and located in the center of the grid, clearly adopting the patterns shape. There are no cells growing in passivated areas.

Most cells adhere to passivated areas of the grid after washing (Figure 13; step 9i).

### Potential solution

• Decrease the concentration of the fibronectin coating solution.
• The passivation is not perfect. Wash earlier as it gives cells less time to adhere.
• Prepare and use fresh stock of the PLL-g-PEG passivation agent.
• Decrease the concentration of your ECM component.

### Problem 7

You observe cell clumps after seeding and washing on your micropatterned grids (step 9i).

### Potential solution

• Your seeding density is too high. Decrease the amount/concentration of the cell suspension used for seeding.
• Your strained cells are already agglomerating in the Falcon tube. You need to shake the Falcon tube occasionally to avoid sedimentation.
• Decrease the time between straining the cells and seeding them onto the grid.
• Be more rigorous with washing to get rid of only peripherally attached cells to the pattern.
• Decrease the size of your pattern.

### Problem 8

The cells do not adhere to the patterned areas (step 9i).

### Potential solution

• Use a fluorophore conjugated ECM component to visualize the pattern and assess its quality.
• Use a different ECM component or increase its concentration.
• Increase the UV irradiation time in the photopatterning process.
• Use a different grid type.
• Assure the cells vitality before seeding.
• Optimize the conditions for cell detachment. Harsh conditions influence the cells’ ability to attach and grow on grids.

### Problem 9

After an initial attachment of the cells to the patterned areas have been observed, they start to round up and detach again (step 9k).

### Potential solution

• Your cell type is not suitable for idle growth over longer time scales.
• Use a different ECM component in your pattern.
• Investigate other patterns in size and shape.
• Use a different grid type.
• Use conditioned media to cultivate the cells on grids in the ibidi chamber slide to decrease cell stress.
• Carefully evaluate the timing when cells look optimal on the grid. If your experiment allows, perform the plunge-freezing then.

### Problem 10

The grid sticks to the plunge-freezers tweezers and you can’t put the grid into the grid box (step 37).

### Potential solution

• Thoroughly submerge the just vitrified grid in LN2 before putting it into the grid box to wash off ethane/propane that sticks to and solidifies between the interface of grid and tweezer causing them to stick together.
• Only grab the far edge of the grid to minimize the contact surface of grid and tweezer, when setting up the grid in the plunge freezer apparatus.

### Problem 11

You can’t see the grid box slots for the grids in the LN2 (step 37).

### Potential solution

• Mark the numbers and the slots outline of the grid box with a fine liner.

### Problem 12

No cells but only ‘footprints’ are visible in the cryoFIB-SEM (step 49).

### Potential solution

• After vitrification, the grid was bend during handling and the cells broke off. Try to handle the grid more carefully.
• The blotting was too long and caused the cells to deflate. Blot shorter.

### Problem 13

The ice is too thick (step 49).

### Potential solution

• Increase blotting time in the plunge freezing process.
• Put a drop of medium on top of the grid prior to blotting. This drop assures capillary forces can pull the moisture into the blotting paper.
• When plunge freezing, thoroughly submerge the just vitrified grid in LN2 before putting it into the grid box to wash off ethane/propane that could stick to and solidify on your grid.

### Problem 14

You observe huge domes of cells on micropatterned grids in the cryoFIB-SEM (step 49).

### Potential solution

• Your pattern is too small or of inappropriate shape for the selected cell line. Increase the pattern size and/or try a different pattern.
• Decrease your seeding density to avoid having multiple cells in one pattern.
• Your cells are clumping together. Use a cell strainer to promote single cells.

### Problem 15

Lamellae are unstable and start to bend (step 57).

### Potential solution

• Mill micro-expansions joints (Wolff et al., 2019).
• Reduce the milling current in the final steps of FIB-milling.
• Add more steps into your milling pattern.

### Problem 16

Patches of hexagonal or cubic ice are visible in the lamella (step 67).

### Potential solution

• Cells that grow in clumps tend to vitrify poorly. Target single cells only.
• Infected cells are round due to sickness. Ideally, one would go for smaller cell lines.
• Cells can be put on a low-FBS diet, which will reduce their size. Conditions to play with would be the starting time of the diet (prior to infection or when starting infection) and the percentage of FBS that is still minimally required to grow the cells without negatively impacting the experiment.
• Incubate the cells right before plunge freezing for 1–5 min in GM supplemented with 10% glycerol. Glycerol serves as a cryo-protectant and has been shown to improve the vitrification of cellular samples (see Bäuerlein et al., 2017).

### Problem 17

Ice clumps cover the view through the lamella (step 67).

### Potential solution

• Tilt the stage from the maximum to minimum range. This sometimes successfully moves the ice contamination off the lamella.

## Resource availability

### Lead contact

Further information and requests for resources and reagents should be directed to and will be fulfilled by the lead contact, Linda E. Franken (linda.franken@cssb-hamburg.de).

### Materials availability

All resources, materials, and software have been bought and are commercially available, except for the FIB-milling autogrids (Schaffer et al., 2015) and the Manual Plunger (Rigort et al., 2010 and Dobro et al., 2010), which have been custom made.

Requests for materials can be made to the lead contact. In principle, the protocol is intended to be able to stand on its own and most materials or equivalents there off are commercially available.

### Data and code availability

No data or code was produced in/for this study.
